# A Multi-Omics Study Revealing the Metabolic Effects of Estrogen in Liver Cancer Cells HepG2

**DOI:** 10.3390/cells10020455

**Published:** 2021-02-20

**Authors:** Minqian Shen, Mengyang Xu, Fanyi Zhong, McKenzie C. Crist, Anjali B. Prior, Kundi Yang, Danielle M. Allaire, Fouad Choueiry, Jiangjiang Zhu, Haifei Shi

**Affiliations:** 1 Department of Biology, Miami University, Oxford, OH 45056, USA; shenm@miamioh.edu (M.S.); xum3@miamioh.edu (M.X.); cristme@mail.uc.edu (M.C.C.); priorab@miamioh.edu (A.B.P.); allairdm@miamioh.edu (D.M.A.); 2 Department of Chemistry and Biochemistry, Miami University, Oxford, OH 45056, USA; zhongf@miamioh.edu (F.Z.); yangk4@miamioh.edu (K.Y.); 3 Department of Human Sciences, College of Education and Human Ecology, Columbus, OH 43210, USA; choueiry.2@buckeyemail.osu.edu; 4 James Comprehensive Cancer Center, The Ohio State University, Columbus, OH 43210, USA

**Keywords:** HepG2 cells, estradiol, estrogen receptor, genomics, metabolomics, gene–metabolite interaction, glycolysis, oxidative phosphorylation, amino acid metabolism

## Abstract

Hepatocellular carcinoma (HCC) that is triggered by metabolic defects is one of the most malignant liver cancers. A much higher incidence of HCC among men than women suggests the protective roles of estrogen in HCC development and progression. To begin to understand the mechanisms involving estrogenic metabolic effects, we compared cell number, viability, cytotoxicity, and apoptosis among HCC-derived HepG2 cells that were treated with different concentrations of 2-deoxy-d-glucose (2-DG) that blocks glucose metabolism, oxamate that inhibits lactate dehydrogenase and glycolysis, or oligomycin that blocks ATP synthesis and mitochondrial oxidative phosphorylation. We confirmed that HepG2 cells primarily utilized glycolysis followed by lactate fermentation, instead of mitochondrial oxidative phosphorylation, for cell growth. We hypothesized that estrogen altered energy metabolism via its receptors to carry out its anticancer effects in HepG2 cells. We treated cells with 17β-estradiol (E2), 1,3,5-tris(4-hydroxyphenyl)-4-propyl-1H-pyrazole (PPT) an estrogen receptor (ER) α (ERα) agonist, or 2,3-bis(4-hydroxyphenyl)-propionitrile (DPN), an ERβ agonist. We then used transcriptomic and metabolomic analyses and identified differentially expressed genes and unique metabolite fingerprints that are produced by each treatment. We further performed integrated multi-omics analysis, and identified key genes and metabolites in the gene–metabolite interaction contributed by E2 and ER agonists. This integrated transcriptomic and metabolomic study suggested that estrogen acts on estrogen receptors to suppress liver cancer cell growth via altering metabolism. This is the first exploratory study that comprehensively investigated estrogen and its receptors, and their roles in regulating gene expression, metabolites, metabolic pathways, and gene–metabolite interaction in HCC cells using bioinformatic tools. Overall, this study provides potential therapeutic targets for future HCC treatment.

## 1. Introduction

Hepatocellular carcinoma (HCC) is one of the most malignant common type of primary liver cancers, whose incidence and mortality have been increasing worldwide [1,2]. The findings from epidemiological studies indicate that people with metabolic diseases have significantly increased risk for HCC [3], which suggests that metabolic defects may serve as a trigger for developing HCC. Additionally, the risk for men to develop HCC is 2-5 folds as for women across different regions of the world [4,5,6,7]. For example, the overall HCC incidence in the United Kingdom increased 2.5-fold between 1993 and 2017, which included a greater increase in men (from five to 14 per 100,000) than in women (from three to six per 100,000) [8]. This sex disparity suggests that estrogen may play a protective role in HCC carcinogenesis [9,10]. Estrogen acts on estrogen receptors (ER) ERα and ERβ expressed in liver cancer specimen tissues from HCC patients [11,12]. 17β-Estradiol (E2), a primary and bioactive form of estrogen in non-pregnant premenopausal females, and its specific ER agonists suppress HCC cell proliferation and promote cell apoptosis in vitro [13,14]. Other studies have demonstrated that estrogen suppresses tumor growth and fibrosis of HCC progression in vivo [15,16].

Altered glucose and lipid metabolism is a common feature of many types of cancer. The most common source of energy production in healthy cells is via mitochondrial oxidative phosphorylation (OXPHOS). Different from healthy cells, cancer cells have increased glucose uptake and higher rates of anaerobic and aerobic glycolysis, followed by lactate fermentation. Cancer cells rapidly grow when comparing to the blood vessels nourishing them; consequently, hypoxia occurs in cancer cells, due to inadequate oxygen being acquired. Rather than producing ATP via mitochondrial OXPHOS as normal cells do, cancer cells tend to use anaerobic glycolysis in the deficiency of oxygen, aerobic glycolysis in the presence of oxygen, and the pentose phosphate pathway parallel to glycolysis [17]. Both of the metabolic pathways convert pyruvate to lactic acid catalyzed by lactate dehydrogenase, and they are followed by lactic acid fermentation in the cytosol. The pathways serve as the primary source of ATP, a phenomenon that is known as the Warburg effect [18,19].

The most commonly studied human HCC-derived cell line in metabolic research is the HepG2 cell line. In general, viral infection does not occur during the development or progression of metabolic disease-induced liver cancers, as hepatitis B virus and hepatitis C virus do not replicate in HepG2 cells. In contrast, many liver cancer cell lines, including HA22T/VGH, Huh7, and Hep3B, are host cells that support the replication of hepatitis viruses [20,21,22]. The HepG2 cell line was studied to avoid confounding factors, such as viral infection. There are many unknowns about HepG2 regarding the regulation of its energy metabolism. First, it is unclear whether the usage of glucose in HepG2 cells favors glycolysis or OXPHOS. Second, in the context of energy metabolism, the biological roles of E2 and different subtypes of ERs during HCC development are unknown. Although attention has been focused on the regulation of glucose usage, glucose production via gluconeogenesis and glycogenolysis, as well as metabolism of fatty acids and amino acids, also influence HCC development. Third, the relationship between metabolic gene expression, genomic pathways, metabolite profiling, and metabolomic pathways has not been established.

In this study, we first determined the dominant metabolic pathway that is utilized by HepG2 cells using 2-deoxy-d-glucose (2-DG), sodium oxamate, and oligomycin. 2-DG blocks glucose metabolism. Sodium oxamate inhibits lactate dehydrogenase that converts pyruvate to lactic acid, thus inhibiting the glycolysis pathway. Oligomycin blocks the mitochondrial proton channel, which uncouples ATP synthesis from electron transport, thus inhibiting mitochondrial OXPHOS. Subsequently, we tested whether E2 regulated metabolism by acting on ERs in HepG2 cells, using ERα-specific agonist 1,3,5-tris(4-hydroxyphenyl)-4-propyl-1H-pyrazole (PPT) and ERβ-specific agonist 2,3-bis(4-hydroxyphenyl)-propionitrile (DPN). The genes that were upregulated and downregulated in response to E2, PPT, or DPN treatment were identified using RNA sequencing (RNA-Seq) as an approach for genome-wide expression profiling. The profiling results were analyzed by Kyoto Encyclopedia of Genes and Genomes (KEGG) functional pathway Gene Ontology (GO) enrichment analysis. The expression of genes encoding key enzymes that are involved in glucose and lipid metabolism identified by RNA-Seq was measured. Furthermore, high and low abundances of metabolites from treated HepG2 cells were detected while using an optimized method of high-pressure liquid chromatography (HPLC) that was coupled with a mass spectrometry (MS) -based targeted metabolic profiling approach to analyze the metabolomes. In order to connect the systematic changes of transcriptome and metabolome induced by E2 and ER antagonists, we performed multi-omics analyses that integrated transcriptome and metabolome findings and identified key genes and metabolites in gene–metabolite interaction, a more advanced approach than transcriptome profiling analysis alone. To our knowledge, this is the first study with high novelty that has explored the impact of estrogen and its receptors on metabolic gene expression, metabolites, metabolic pathways, and gene–metabolite interaction in HCC-derived HepG2 cells using bioinformatic tools. The identified metabolic genes and pathways that were impacted by estrogen and different ERs could pave the way for a future comprehensive understanding of the metabolic effects of estrogen in HCC progression and potential targets for treating HCC.

## 2. Materials and Methods

### 2.1. Cell Culture

The HepG2 cell line was obtained from American Type Culture Collection (ATCC, Manassas, VA, USA) and authenticated by ATCC with short tandem repeat genotyping analysis. HepG2 cells in early passages were used to maintain a close resemblance of the original HCC cancerous cells. This avoided the potential for culture-induced cell instability and selective growth of rapidly growing cells with greater molecular abnormalities. Although the expression of ERα and ERβ in HepG2 cells has been reported in our [13] and others’ studies [23,24,25,26], some studies failed to detect ERα gene expression [27]. It is possible that ERα gene expression is diminished over long-term cell culture [23]. Using Western blot analysis, the expression of ERα and ERβ was detected in protein that was extracted from tested HepG2 cells.

HepG2 cells were maintained in tissue culture dishes (diameter 100 mm) in phenol red-free Dulbecco’s Modified Eagle Medium (Fisher Scientific, Waltham, MA, USA) that was supplemented with 10% (*v*/*v*) heat-inactivated and charcoal-stripped FBS (Fisher Scientific), 1% antibiotics of 50 U/mL penicillin, and 50 µg/mL streptomycin (Invitrogen, Grand Island, NY, USA) at 37 °C and 5% CO_2_/95% air. When the initial cells (1 × 10^5^/mL) became ~70% confluent, the cells were starved with medium low in serum (0.1% *v*/*v* FBS) for 16 h before treatments.

### 2.2. Cell Treatment

The cells were treated with 2-DG (0–10 mM), sodium oxamate (0–50 mM), or oligomycin (0–1.0 µg/mL; Santa Cruz, Dallas, TX, USA) for 24 h to test major metabolic pathways that are utilized by HepG2 cells. Each chemical was dissolved in DMSO and further diluted to final concentrations. Cells were treated with E2 (Sigma-Aldrich, St. Louis, MO, USA), ERα agonist PPT (Fisher Scientific), or ERβ agonist DPN (Fisher Scientific) for 48 h to examine effects of E2 and ERs. E2 and ERs were dissolved to 1 μM in DMSO and diluted to 10 nM in culture medium. Vehicle DMSO was the control treatment. The concentration ranges of these chemicals are commonly used in liver cancer research and our previous studies [13,14,28,29,30].

### 2.3. Cell Number, Cytotoxicity, Viability, and Apoptosis

Confluent cells (1 × 10^4^/mL) were seeded in tissue culture dishes (diameter 60 mm) and then treated with 2-DG, sodium oxamate, or oligomycin. The growth of HepG2 cell was assessed using light microscopy. The cell numbers were measured using a TC10 automated cell counter (Bio-Rad, Hercules, CA, USA), which counted cells with diameters between 6 and 50 µm. HepG2 cells (~500 cells/well) were seeded in 96-well cell culture plates, treated with 2-DG, sodium oxamate, or oligomycin, and then measured in triplicates in order to assess cytotoxicity, viability, and apoptosis. Viability indicated by live-cell protease activity, cytotoxicity indicated by dead-cell protease activity, and caspase activation-related apoptosis were evaluated using ApoTox-Glo Triplex Assay (Promega, Madison, WI, USA).

### 2.4. ERα and ERβ Protein Detection by Western Blot Analysis

The HepG2 cells were trypsinized, proteins were extracted, and protein lysates were separated using gel electrophoresis and then transferred to nitrocellulose membranes (Bio-Rad). ERα and ERβ (1:200; Santa Cruz, Dallas, TX, USA) and β-actin (a housekeeping protein control; 1:1000; Cell Signaling, Danvers, MA, USA) were detected by standard immunoblotting and chemiluminescence (Amersham ECL Prime, GE Healthcare, Chicago, IL, USA). Protein bands and a protein ladder with a mid-range molecular weight (a molecular size marker; Abcam, Cambridge, MA, USA) were visualized using an Odyssey Infrared Imaging System (LI-COR, Lincoln, NE, USA). Western blot analysis detected the expression of ERα and ERβ in HepG2 cells (Supplemental Figure S1).

### 2.5. Transcriptome Functional Analysis

The RNA-Seq using high-quality RNA samples with RNA integrity numbers above 9, isolated from ~10^6^ HepG2 cells treated with vehicle, E2, PPT, or DPN using RNeasy Mini Kits (Qiagen, Foster City, CA, USA), was conducted at the University of Cincinnati Genomics, Epigenomics, and Sequencing core facility, according to the standardized protocols that were developed by the facility [14]. Briefly, cDNA was converted from1 μg isolated total RNA using a cDNA synthesis kit (Bio-Rad). The cDNA libraries were amplified while using the universal and index-specific primer.

The purified library was checked for quality and yield using a DNA high sensitivity chip and a Bioanalyzer (Agilent, Santa Clara, CA, USA). The library concentration for clustering was measured using PCR and a Kapa Library Quantification kit (Kapa Biosystems, Woburn, MA, USA). The libraries were then pooled for clustering and sequenced as single-end 50 bp on an Illumina HiSeq 2000 system (Illumina, San Diego, CA, USA), which retrieved ~25 million sequence reads from each sample. The sequences were deposited at Gene Expression Omnibus with accession number GSE112983, being aligned to the human genome (ENSEMBL GRCh38.p10) and analyzed for differentially expressed genes using RStudio DESeq2 package. DAVID functional annotation (https://david.ncifcrf.gov/ (accessed on 19 February 2021)) bioinformatics analysis was performed to identify significant KEGG functional pathways comprised of differentially expressed genes and to categorize the pathways containing similar associated genes into the same groups. A *p* value for each group of KEGG pathways and its adjusted *p* value were calculated in E2-, PPT-, or DPN-treated cells as compared to the cells receiving control treatment, with the adjusted *p* value < 0.05 considered to be significant.

### 2.6. Reverse Transcription Quantitative PCR (qPCR)

KEGG pathway analysis revealed many metabolic pathways. We then measured metabolic genes of interest including glucose transporter 2 (*GLUT2*), which takes up glucose into hepatocytes; 6-phosphofructokinase (*6PFK*) and pyruvate kinase (*PK*), which encode two rate-limiting enzymes in glycolysis; cytochrome c oxidase subunit 6B (*COX6B*) for OXPHOS; glycogen phosphorylase liver form (*PYGL*) for glycogenolysis; glycogen synthase 2 (*GYS2*) for glycogen synthesis; phosphoenolpyruvate carboxykinase cytoplasmic form (*PEPCK1*) for gluconeogenesis; transcriptional factor peroxisome proliferator-activated receptor (PPAR) gamma coactivator 1 alpha (*PGC1A*), which regulates *PEPCK1* expression; PPAR gamma (*PPARG*) and sterol regulatory element-binding transcription factor 1 (*SREBP1C*), which encodes two lipogenic transcriptional factors; and, acetyl-CoA carboxylase (*ACC*) and fatty acid synthase (*FAS*), which encode enzymes for fatty acid biosynthesis. β-Actin (*ACTB*) mRNA levels were similar among groups and *ACTB* was used as a reference gene. The qPCR was carried out in triplicates on a qPCR instrument (Bio-Rad CFX96) using primers (Integrated DNA Technologies, San Jose, CA, USA; Table 1) and SYBR green master mixes. Gel electrophoresis and melt curve analysis were used to confirm the amplified PCR products. The expressions were normalized to *ACTB* and presented using control group as 100%.

### 2.7. HPLC-MS/MS Targeted Metabolite Analysis

Metabolites were extracted from ~10^6^ cells that received the same treatment of vehicle, E2, PPT, or DPN as RNA-Seq analysis following the established chromatography protocol [31,32]. In brief, the cells were homogenized with phosphate-buffered saline, extracted in 250 μL cold methanol, and then mixed with 50 μL isotopically labeled spiking solution with a mixture of C^13^ amino acids and C^13^ lactate as internal standards (Cambridge Isotope Laboratories Inc., Tewksbury, MA, USA). The mixture was incubated at −20 °C for 20 min. and then centrifuged at a speed of 14,000 rpm for another 20 min. The supernatant (150 μL) was then collected, dried, reconstituted in mobile phase with a mixture of HPLC-MS grade water, acetonitrile, ammonium acetate, and acetic acid (Fisher Scientific), and it was measured according to their corresponding standard chemical compounds (Sigma-Aldrich and IROA Technologies, Boston, MA, USA).

Targeted metabolite profiling was operated according to our validated method [31,32,33,34,35], using an Ultimate 3000 HPLC that was coupled with a TSQ Quantiva triple quadrupole MS (Thermo Fisher Scientific, Waltham, MA, USA) installed with a hydrophilic interaction chromatography column (Waters Corporation, Milford, MA, USA). The total separation for positive and negative ionization modes was 20 min. at a flow rate of 0.300 mL/min., with the HPLC gradient separation running for 11 min, followed by a 9-min wash to avoid potential carryover. Targeted data acquisition was performed together with multiple authentic standards in selected reaction monitoring (SRM) mode. The instrumentation method with built-in retention time and SRM transition information was used to detect the targeted metabolites in unknown samples.

All 221 measured metabolites that represented key metabolites of interest from relevant metabolic pathways [36,37] have been validated in our published work [31,32,33,34,35] and they are consistent with others’ studies. The analyzed metabolites were determined by the detection ability and measurement reliability. Based on the low (<15%) average inter-assay coefficient of variation for the quality control samples, our targeted metabolic profiling approach had an excellent reproducibility. All of the recorded mass spectra were manually inspected using the Quanbrowser module of Xcalibur (V 4.0, Thermo Fisher Scientific, Waltham, MA, USA). The MS data sets were normalized by the cell number counts at the point of metabolite extraction. JMP Pro12 (SAS Institute, Cary, NC, USA) was used for statistical analysis. Principle components analysis was used to compare metabolic profiles among different groups.

### 2.8. Metabolic Pathways Analysis

All of the detected metabolites were analyzed for metabolic pathways to explore the metabolic impact of each treatment on HCC cells using MetaboAnalyst 4.0 computational platform (http://www.metaboanalyst.ca/ (accessed on 19 February 2021)), to achieve a broad coverage of extensive metabolic networks [38]. Metabolic pathway impact analyses were conducted via introducing an individual metabolite into the context of connected metabolic pathway networks in the KEGG database in order to obtain the number of identified metabolites (hits) of each pathway with a known number of total composed metabolites. A raw *p* value for each metabolic pathway with a group of functionally associated metabolites, and its adjusted *p* values, were calculated in E2- or ER agonist-treated cells when comparing to the cells receiving control treatment, and adjusted *p* values < 0.05 were considered to be significant. False discovery rate (FDR) was used to control for false positives, and an FDR threshold of 0.05 that yields < 5% false positives [39] was applied to identify significantly enriched pathways. Based on the relative importance of individual nodes within the overall network, pathway analysis also computed the importance of each identified metabolite in pre-defined metabolic pathways. Additionally, pathway analysis computed a pathway impact score as the sum of the importance measures of identified metabolites divided by the total sum of the importance measures of all the identified and unidentified metabolites in the pathway. The importance of a given pathway relative to a global metabolic network was estimated by its pathway impact score. The pathways with impact scores > 0.05 were considered to be important metabolic pathways.

### 2.9. Multi-Omics Integration Analysis

Multi-omics integration analyses using transcriptome and metabolome data were performed using MetaboAnalyst 4.0 Joint Pathway Analysis Module (http://www.metaboanalyst.ca/ (accessed on 19 February 2021)) and Ingenuity Pathway Analysis (Qiagen, Germantown, MD, USA). Gene–metabolite interaction networks were established to show first-order relationships based on MetPriCNet (https://www.metricnet.com/ (accessed on 19 February 2021)) [40], a curated compound interaction database extracted from published literature. The chemical associations for the gene–metabolite and metabolite-metabolite networks were extracted from STITCH (http://stitch.embl.de/ (accessed on 19 February 2021)) [41], ensuring that only highly confident interactions were represented.

### 2.10. Statistical Analysis

Comparisons of cell number, viability, cytotoxicity, apoptosis, gene expression, metabolite levels, and ADP/ATP ratio were used one-way analysis of variance (ANOVA), followed by Tukey’s multiple comparison post hoc test (GraphPad Prism 8; La Jolla, CA, USA), with *p* < 0.05 being considered to be statistically significant.

## 3. Results

### 3.1. Effects of 2-DG, Oxamate, and Oligomycin on Cell Growth

**Figure 1:**
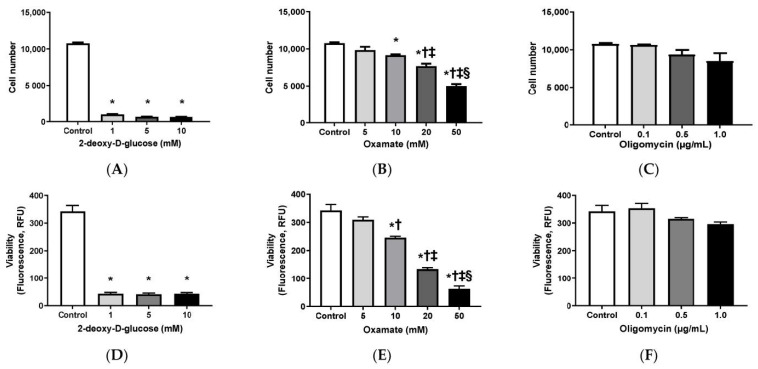

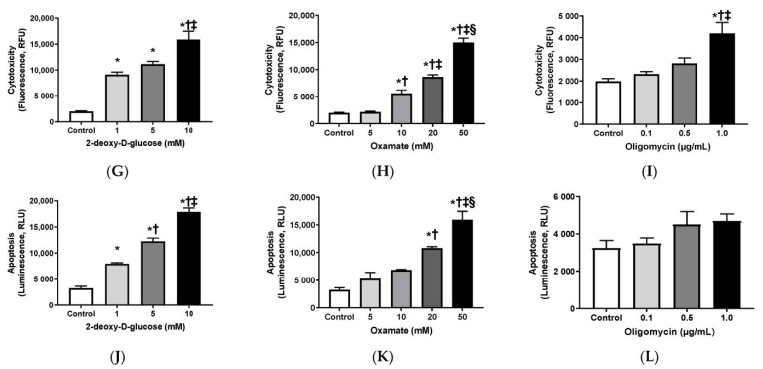
Effects of 2-deoxy-D-glucose, oxamate, and oligomycin on HepG2 cell number and growth. Cell numbers (**A**–**C**), viability (**D**–**F**), cytotoxicity (**G**–**I**), and apoptosis (**J**–**L**) of HepG2 that were treated with different concentrations of 2-deoxy-D-glucose (0–10 mM; G), oxamate (0–50 mM; H), and sodium oxamate (0–1 µg/mL; E). Cell number data and fluorescence or luminescence signal data were represented as mean ± SEM (*n* = 5/group) and analyzed by one-way ANOVA analysis. For Figure 1A,D,G,J, *: Significantly different comparing with control group; †: Significantly different from 1 mM group; and ‡: Significantly different from 5 mM group. For Figure 1B,E,H,K, *: Significantly different comparing with control group; †: Significantly different from 5 mM group; ‡: Significantly different from 10 mM group; and §: Significantly different from 20 mM group. For Figure 2F, *: Significantly different comparing with control group; †: Significantly different from 0.1 µg/mL group; and ‡: Significantly different from 0.5 µg/mL group. (*p* < 0.05).

When compared to the control treatment, 2-DG at all concentrations tested (1, 5, and 10 mM; Figure 1A; Supplemental Figure S2) and oxamate at the higher concentrations tested (10, 20, and 50 mM; Figure 1B; Supplemental Figure S2) significantly decreased the HepG2 cell numbers. Additionally, while 2-DG at different concentrations reduced cell number to a similar extent, oxamate led to a concentration-dependent cell number reduction. In contrast, treatment with 1.0 µg/mL or lower concentrations of oligomycin did not significantly change the cell numbers (Figure 1C; Supplemental Figure S2).

The cell viability mirrored the cell count results. Specifically, 2-DG at all concentrations tested (Figure 1D) and oxamate concentration-dependently (Figure 1E) reduced cell viability when compared with the control treatment; however, no difference in viability was found among the groups treated with oligomycin (Figure 1F). In general, cells that were treated with 2-DG (Figure 1G), oxamate (Figure 1H), and oligomycin (Figure 1I) showed a concentration-dependent increase in cytotoxicity. Additionally, 2-DG (Figure 1J) and oxamate (Figure 1K) increased apoptosis in a concentration-dependent manner. In contrast, the highest concentration of the oligomycin treatment tested significantly increased cytotoxicity (Figure 1I), but did not significantly affect viability (Figure 1F) or apoptosis (Figure 1L). The HepG2 cell number and viability were suppressed, while cytotoxicity and apoptosis were elevated, when glucose metabolism was blocked by 2-DG and glycolysis was blocked by oxamate. Blocking OXPHOS by oligomycin did not significantly affect the cell number, viability, or apoptosis, but did increase cytotoxicity when HepG2 were treated with the highest concentration of oligomycin, which suggested that HepG2 cell growth dominantly relied on glycolysis, rather than OXPHOS.

### 3.2. Effects of E2 and ER Agonists on Transcriptome Functional Pathways

In order to explore potential mechanisms underlying protective effects of estrogen on HCC development, the comprehensive global transcriptome profiles regulated in response to E2, ERα specific agonist PPT, or ERβ specific agonist DPN were generated using RNA-Seq as an approach for genome-wide expression profiling and then analyzed by KEGG functional pathway enrichment analysis.

**Figure 2:**
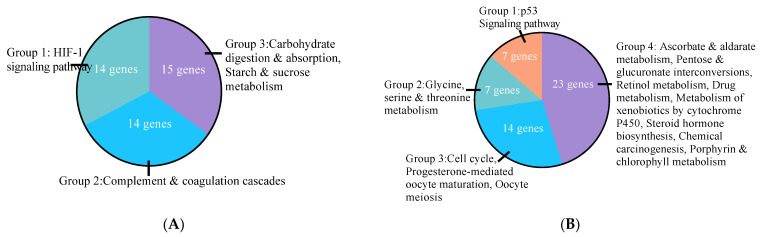

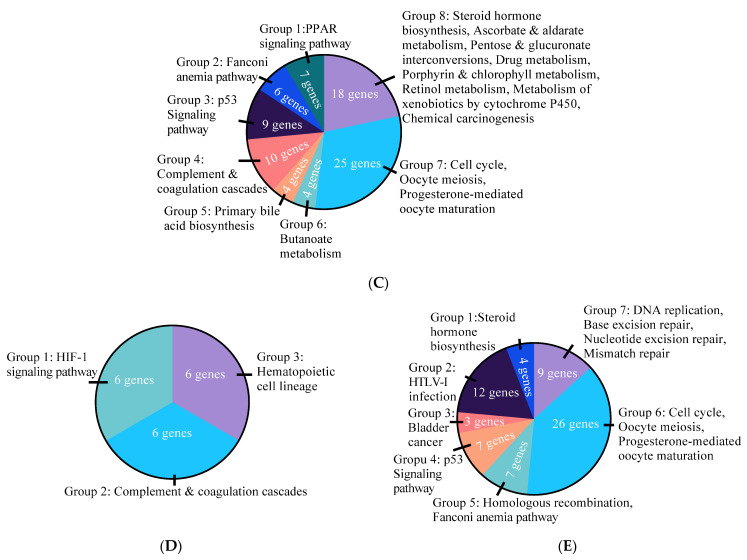
Effects of estradiol, estrogen receptor α (Erα) specific agonist 1,3,5-tris(4-hydroxyphenyl)-4-propyl-1H-pyrazole (PPT), and ERβ specific agonist 2,3-bis(4-hydroxyphenyl)-propionitrile (DPN) on HepG2 cell Kyoto Encyclopedia of Genes and Genomes (KEGG) functional pathway Gene Ontology (GO) enrichment analysis of differentially expressed genes analyzed using RNA sequencing reads deposited at Gene Expression Omnibus (accession number GSE112983). Pathways containing similar associated genes were categorize into the same groups. (**A**) Upregulated genes and KEGG pathways GO analysis of HepG2 treated with estradiol. (**B**) Downregulated genes and KEGG pathways GO analysis of HepG2 treated with estradiol. (**C**) Downregulated genes and KEGG pathways GO analysis of HepG2 treated with PPT. (**D**) Upregulated genes and KEGG pathways GO analysis of HepG2 treated with DPN. (**E**) Downregulated genes and KEGG pathways GO analysis of HepG2 treated with DPN.

E2 had the most evident impact on cell metabolism and function among the treatment groups when compared to the control group, with 956 upregulated genes being associated with KEGG pathway groups that are linked to hypoxia-inducible factor-1 (HIF-1) signaling, complement and coagulation cascades, and carbohydrate digestion and absorption, and starch and sucrose metabolism (Figure 2A; Table 2; Supplemental Table S1). The transcriptome analysis revealed 380 downregulated genes by E2 treatment, which were associated with KEGG pathway groups that were linked to p53 signaling pathway, glycine, serine and threonine metabolism, cell cycle and progesterone-mediated oocyte maturation, and oocyte meiosis. Ascorbate and aldarate metabolism, pentose/glucuronate interconversions, retinol metabolism, drug metabolism, metabolism of xenobiotics by cytochrome P450, steroid hormone biosynthesis, chemical carcinogenesis, and porphyrin and chlorophyll metabolism were also included (Figure 2B; Table 2; Supplemental Table S2).

The transcriptome analysis revealed 242 upregulated genes by ERα agonist PPT without any significantly associated KEGG pathway. PPT-treated cells downregulated 397 genes that were associated with KEGG pathways linked to the same groups of p53 signaling, cell cycle, and metabolisms as E2 treatment, and additional PPAR signaling pathway, Fanconi anemia pathway, complement and coagulation cascades, primary bile acid biosynthesis, and butanoate metabolism (Figure 2C; Table 2; Supplemental Table S3).

The transcriptome analysis revealed 254 upregulated and 271 downregulated genes by DPN. The enhanced pathways by ERβ agonist DPN were HIF-1 signaling, complement and coagulation cascades, and hematopoietic cell lineage (Figure 2D; Table 2; Supplemental Table S4). The suppressed pathways by DPN treatment were the same steroid hormone biosynthesis, cell cycle, and p53 signaling pathway as E2 and PPT; with additional pathways in human T-cell leukemia virus type 1 (HTLV-I) infection, bladder cancer, homologous recombination, and Fanconi anemia pathway, as well as DNA replication, base excision repair, nucleotide excision repair, and mismatch repair (Figure 2E; Table 2; Supplemental Table S5).

### 3.3. Effects of E2 and ER Agonists on Metabolic Genes

KEGG pathway analysis of the transcriptome profiles identified by RNA-Seq revealed many metabolic pathways; therefore, the expression of genes encoding key enzymes involved in glucose and lipid metabolism was measured using qPCR.

**Figure 3:**
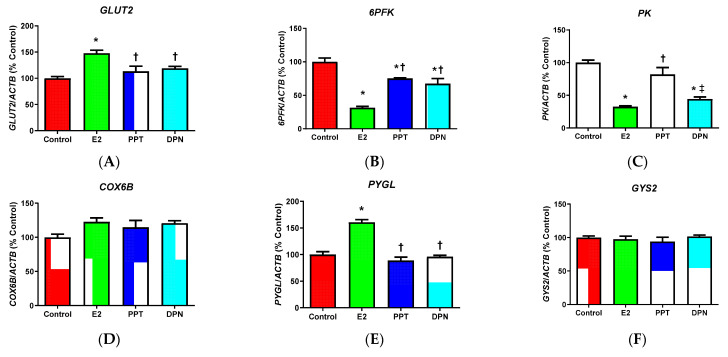

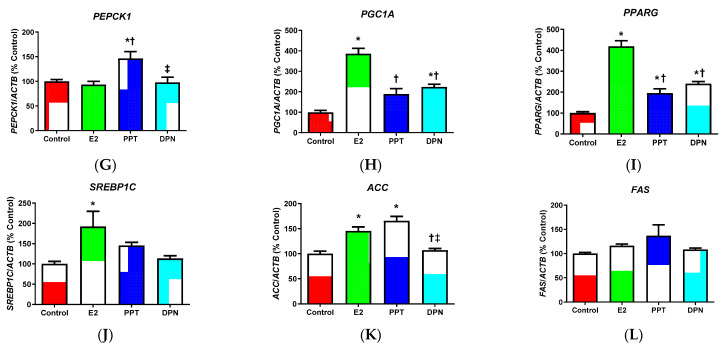
Effects of estradiol (E2), ERα specific agonist PPT, and ERβ specific agonist DPN on HepG2 cell expression of genes involved in energy metabolism. (**A**–**L**) The mRNA levels of (**A**) glucose transporter 2 (*GLUT2*), (**B**) phosphofructokinase-6 (*6PFK*), (**C**) pyruvate kinase (*PK*), (**D**) cytochrome c oxidase (*COX6B*), (**E**) glycogen phosphorylase (*PYGL*), (**F**) glycogen synthase (*GYS2*), (**G**) phosphoenolpyruvate carboxykinase cytoplasmic form (*PEPCK1*), (**H**) peroxisome proliferator-activated receptor (PPAR) gamma coactivator 1 alpha (*PGC1A*), (**I**) PPAR gamma (*PPARG*), (**J**) sterol-regulatory binding protein-1c (*SREBP1C*), (**K**) acetyl-CoA carboxylase (*ACC*), and (**L**) fatty acid synthase (*FAS*) were normalized to reference gene β-actin (*ACTB*) mRNA levels and % of the control group. The data were represented as mean ± SEM (*n* = 5/group) and analyzed by one-way ANOVA analysis. *: Significantly different when comparing to control groups; †: Significantly different comparing to E2 groups; and ‡: Significantly different comparing to PPT groups (*p* < 0.05).

When compared to the control treatment expression of *GLUT2*, which encodes GLUT2, which takes up glucose into hepatocytes, was increased by E2, but not PPT or DPN (Figure 3A), indicating increased glucose uptake into HepG2 cells by E2. The expression of *6PFK* encoding an ATP-dependent glycolysis rate-limiting enzyme was significantly lowered by all treatments, with E2 treatment having the most prominent effects (Figure 3B). The expression of another glycolysis rate-limiting enzyme, *PK*, was significantly reduced by E2 and DPN (Figure 3C). The expression of *COX6B*, an important mitochondrial OXPHOS gene, was not significantly changed by E2 or ER agonist treatment (Figure 3D). The expression of *PYGL* coding a glycogenolysis enzyme that breaks down glycogen in hepatocytes was enhanced by E2 (Figure 3E); whereas, expression of *GYS2*, which is a critical gene for glycogen synthesis, was not significantly altered by any E2 or ER agonist (Figure 3F). Additionally, PPT treatment induced expression of *PEPCK1* (Figure 3G), a gene regulating gluconeogenesis, while E2 and DPN treatments induced expression of *PGC1A* (Figure 3H), a transcriptional factor regulating *PEPCK1* expression to activate gluconeogenesis [42]. The expressions of lipogenic transcriptional factors *PPARG* and *SREBP1C*, which function to activate fatty acid biosynthesis, were also enhanced. Specifically, *PPARG* expression was enhanced by E2 and ER agonists, with E2 having more pronounced effects than PPT and DPN treatments (Figure 3I); and, *SREBP1C* expression was enhanced by E2 (Figure 3J). Furthermore, genes encoding two additional important enzymes for fatty acid biosynthesis *ACC* and *FAS* were measured. E2 and PPT both significantly induced the expression of *ACC* as compared with control and DPN (Figure 3K), but no significant difference in *FAS* expression was detected in any groups (Figure 3L).

### 3.4. Effects of E2 and ER Agonists on Metabolic Profiles

**Figure 4:**
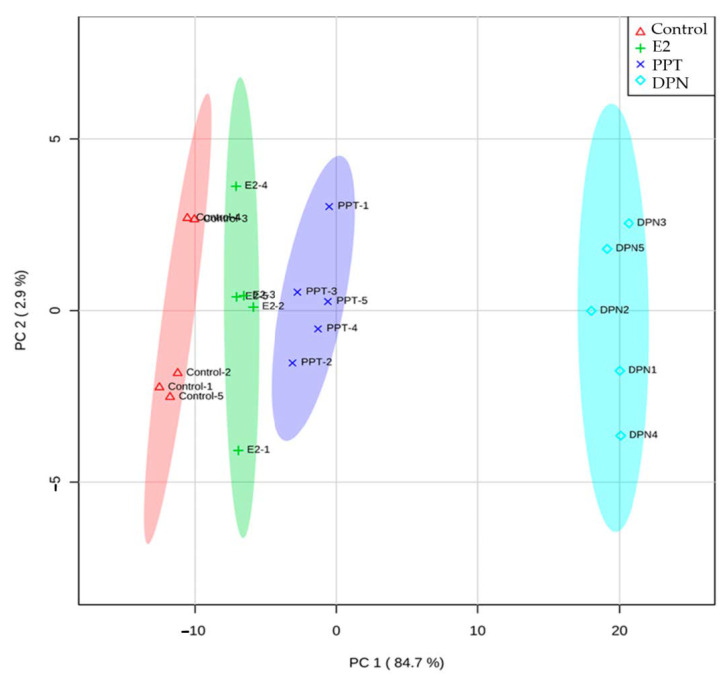
Principal component analysis showing metabolic profile-based separation of HepG2 cells treated with control, estradiol (E2), ERα specific agonist PPT, and ERβ specific agonist DPN. Each symbol represents one biological replicate, the shading area indicates the 95% confidence interval of grouping.

A metabolomic analysis was performed because differential expressions of metabolic genes were detected by the treatments. Among the 221 metabolites tested, 174 metabolites were detected in all of the samples and compared for relative concentrations. Clear patterns were shown in the heatmap, which indicated greater levels of metabolites in the E2 and ER agonist groups, with DPN treatment showing the greatest abundance (Supplemental Figure S3). The principal component analysis score plot indicated a separation of the targeted metabolic profiles among all four groups (Figure 4), which suggested that E2, PPT, and DPN treatments exhibited unique metabolic fingerprints that were different from the control treatment.

**Figure 5:**
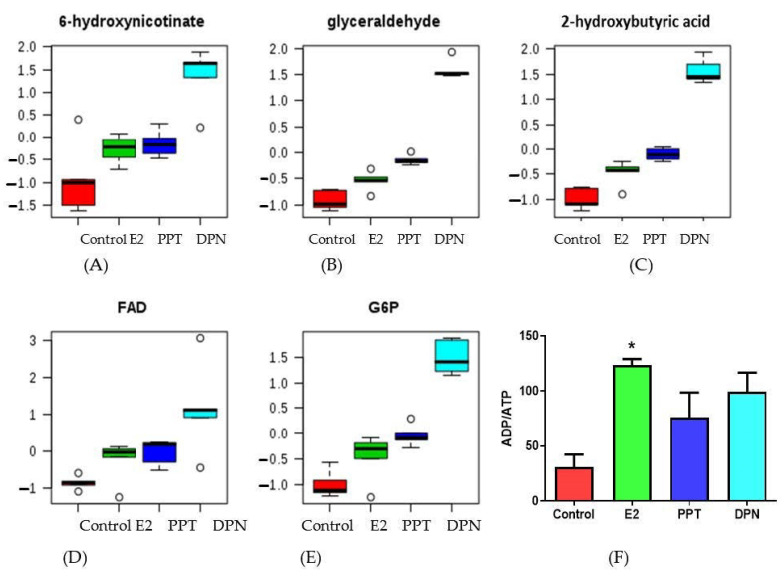
Effects of estradiol (E2), ERα specific agonist PPT, and ERβ specific agonist DPN on levels of representative metabolites of glucose and lipid metabolism and ADP/ATP ratio. (**A**–**E**) Relative units of the levels of metabolites that were identified different from control group. The representative metabolites included 6-hydroxynicotinate (**A**), glyceraldehyde (**B**), 2-hydroxybutyric acid (**C**), flavin adenine dinucleotide (FAD; **D**), and glucose-6-phosphate (G6P; **E**). Data (mean ± standard deviation) were normalized and auto-scaled using Metaboanalyst program. The analysis process rendered negative unit levels of metabolites in some groups. Boxes presented 25–75% of normalized values with medians indicated by horizontal lines within boxes, error bars indicated 5–95% of normalized values, and data points that were <5% or >95% of normalized values were indicated using open circles. (**F**) The ADP/ATP ratio data were represented as mean ± SEM. Data were analyzed by one-way ANOVA analysis. *: Significantly different comparing to the control group (*p* < 0.05).

We confirmed a few representative metabolites that contributed to the separation of the metabolic profiles among the groups. When comparing to the control treatment, the E2 and ER agonists induced levels of 6-hydroxynicotinate (Figure 5A) from nicotinate and nicotinamide metabolism; glyceraldehyde (Figure 5B), an intermediate of glucose metabolism; 2-hydroxybutyric acid (Figure 5C), a product in amino acid catabolism; flavin adenine dinucleotide (FAD, Figure 5D), which is involved in several important metabolic enzymatic reactions; and, a glycolysis intermediate glucose-6-phosphate (G6P, Figure 5E). DPN had the most marked effects in increasing all five metabolites. Additionally, E2 significantly increased, whereas PPT and DPN had a trend to increase the ADP/ATP ratio (Figure 5F).

### 3.5. Effects of E2 and ER Agonists on Metabolic Pathways

The detected metabolites were analyzed for metabolic pathways between each treatment group and the control group (Supplemental Tables S6–S8; Table 3). All of the E2 and ER agonists commonly impacted amino acid metabolic pathways (i.e., tyrosine, tryptophan, histidine, glycine/serine/threonine, arginine/proline, and taurine/hypotaurine) and carbohydrate metabolic pathways (i.e., amino sugar/nucleotide sugar, pyruvate, TCA cycle, and glycolysis and gluconeogenesis). E2 and DPN also significantly impacted cofactors and vitamin metabolism involving one carbon pool by folate. Both of the ER agonists significantly impacted amino acid metabolic pathways (i.e., cysteine/methionine, beta-alanine, arginine biosynthesis, alanine/aspartate/glutamate, and glutathione); carbohydrate metabolic pathways (i.e., glyoxylate/dicarboxylate, starch/sucrose, and pentose/glucuronate interconversions); glycerophospholipid metabolism; cofactors and vitamins metabolic pathways (i.e., vitamin B6 and nicotinate/nicotinamide); nucleotide pyrimidine and caffeine metabolic pathways; and, translation processing involving aminoacyl-tRNA biosynthesis. DPN alone also affected inositol phosphate and biotin metabolism (Table 3).

### 3.6. Effects of E2 and ER Agonists on Gene–Metabolite Interaction

**Figure 6:**
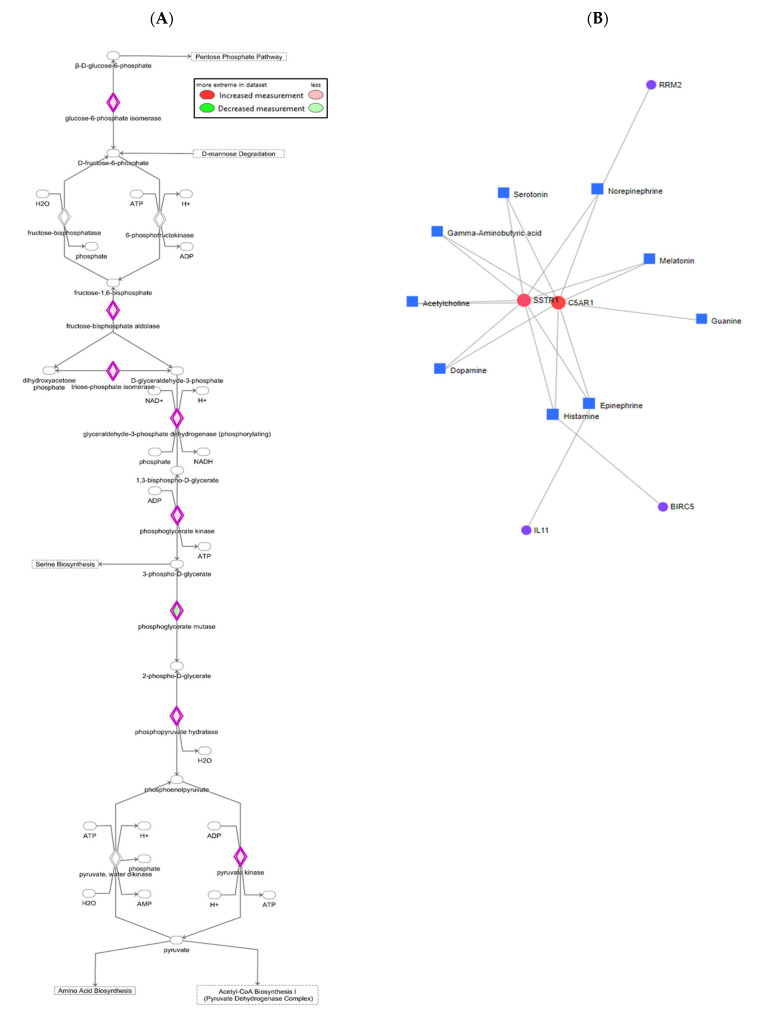
Gene–metabolite interaction revealed using multi-omics analysis. (**A**) Enzymes in glycolysis pathway altered by estradiol treatment. (**B**) Commonly altered genes and metabolites among all three networks (see Supplemental Figure S4) of HepG2 cells treated with estradiol, ERα specific agonist PPT, and ERβ specific agonist DPN. Each square node represents one metabolite and each circle node represents one gene. Sizes of nodes are proportional to their degree values, signifying number of their connections to other nodes. Nodes with higher degree values act as hubs in a network. Colors of nodes reflect their betweenness centrality values, signifying number of shortest paths or interactions going through the nodes.

We performed multi-omics integration analyses using powerful bioinformatics tools to connect the systematic changes of transcriptome and metabolome induced by E2 and ER agonists. First, by integrating the transcriptome and metabolome results, IPA identified eight important enzymes of glycolysis pathway that were altered by treatments (Figure 6A), including glucose-6-phosphate isomerase, fructose-bisphosphate aldolase, triose-phosphate isomerase, glyceraldehyde-3-phosphate dehydrogenase, phosphoglycerate kinase, phosphoglycerate mutase, phosphoenolpyruvate hydratase, and pyruvate kinase. This is in agreement with predictions that were made using curated results of the Ingenuity Knowledge Base of IPA. We further evaluated the effect of E2 and ER agonists on transcriptome and metabolome of HepG2 cells using differentially expressed genes that were identified by RNA-Seq and metabolites detected by targeted metabolomics. Interaction maps indicated that 52 genes and 44 metabolites in E2-treated cells (Supplemental Figure S4A), 24 genes and 21 metabolites in PPT-treated cells (Supplemental Figure S4B), and 21 genes and 20 metabolites in DPN-treated cells (Supplemental Figure S4C) were connected based on the reported network in the KEGG database. We then overlapped these three gene–metabolite interaction maps and generated a summary map of 5 genes (i.e., *SSTR1*, *C5AR1*, *RRM2*, *IL11*, and *BIRC5*) (Figure 6B). Somatostatin receptor (SSTR) 1 (*SSTR1*) and complement component 5a (C5a) receptor 1 (*C5AR1*) served as central hubs. The summary map also included nine endogenous metabolites mainly from amino acid metabolic pathways: serotonin, gamma-aminobutyric acid, acetylcholine, dopamine, histamine, epinephrine, guanine, melatonin, and norepinephrine (Figure 6B). The summary map reflected common changes in gene–metabolite interaction that are caused by E2, PPT, and DPN treatments, and provided metabolic and signaling pathways that could be explored in the future.

## 4. Discussion

### 4.1. Summary of Findings

The lower incidence of HCC in women as compared to men could be attributed to the protective roles of estrogen in HCC development and progression. Although there has been considerable progress in demonstrating anticancer effects of estrogen, the mechanisms that are related to metabolic reprogramming that underlie HCC protection remain unclear [43]. We took advantage of transcriptome and metabolome pathway analyses and multi-omics analysis of gene–metabolite interaction to explore potential mechanisms involving metabolic action by estradiol and its receptor agonists. Through these techniques, altered metabolic gene expression and pathways affected by estrogen and its receptor agonists were identified. Key observations included the suppression of fuel usage via glycolysis, pentose/glucuronate interconversions, and altered metabolism of amino acids and fatty acids. Conversely, enhanced fuel storage via glucose uptake, glucose production by means of glycogenolysis and gluconeogenesis, along with lipogenesis by estrogen and its receptor agonists were also observed. All of which could potentially inhibit HepG2 cell growth. ERα and ERβ are reported to interact with each other and they have opposing actions in breast cancer, prostate cancer, and ovarian cancer [44,45]. Future experiments with activation or knockdown of respective ERα and ERβ will be conducted to confirm the functions of individual ERs and the effects of ER agonists, as well as to test the possible interaction between ERα and ERβ.

### 4.2. Metabolism Reprograming in HepG2 Cells

Healthy liver cells perform various essential functions in addition to digestion and detoxification, such as glucose, lipid, and amino acid metabolism, in order to maintain a balance between the storage and utilization of fuels. Energy metabolism reprogramming is essential for the survival and growth of cancer cells. A well-known metabolic alternation of cancer cells is the Warburg effect. Cells become dependent on glycolysis instead of OXPHOS to produce ATP, even in the presence of abundant oxygen. Aerobic glycolysis supplies cancer cells with intermediates that are essential for cell proliferation. It has been reported that higher levels of lactate, the glycolysis end product, are detected in aggressive, drug-resistant, and metastatic cancer cells [46]. Although the Warburg effect is widely recognized, not all cancer cells producing ATP primarily rely on glycolysis. For example, NB4 leukemia cells are sensitive to glucose metabolism inhibitor 2-DG and they are considered to be “glycolytic” cells. THP-1 leukemia cells are 2-DG resistant, but sensitive to OXPHOS inhibitor oligomycin, and they are considered “OXPHOS” cells [47]. Because energy metabolism mechanisms vary in different types of cancer cells, specific metabolic pathways can be used as targets for cancer therapy.

The first experiment of this study indicated that HepG2 cells were sensitive to the treatment of a glucose metabolism blocker, 2-DG, and a lactate dehydrogenase and glycolysis inhibitor, oxamate. These treatments lead to reduced cell numbers and lower viability, but greater markers of cytotoxicity and apoptosis (Figure 1). In contrast, HepG2 cells were resistant to the treatment of oligomycin, an inhibitor for mitochondrial ATP synthase. HepG2 cell growth was profoundly suppressed when glucose metabolism was blocked or when glycolysis was inhibited, but it was not significantly affected when mitochondrial OXPHOS was abolished. This indicates that glycolysis was the dominant metabolic pathway supporting HepG2 cell growth. It is noteworthy that the concentrations tested in HepG2 cells could have very different effects in other cell lines. For example, 1 mM 2-DG blocked cell viability and induced cytotoxicity in liver cancer cells HepG2 in this study. It has been reported that 1 mM 2-DG is toxic in breast cancer cell SUM149 [48], but it is not toxic in other breast cancer cells MDA-MB-231, HCC1937, HDQ-P1, and MCF-7 cells [48,49]. Thus, the findings from this study can be referenced in future studies.

### 4.3. Effects of E2 and ER Agonists on Metabolic Gene Regulation in HepG2 Cells

E2 is known to regulate gene transcription by acting on its nuclear receptors. By investigating the effects of agonists of ERα and ERβ on metabolic genes and metabolites in HepG2 cells, this study is of great interest, as it contributes to the understanding of estrogen actions via different ER isoforms in liver cancer development [10]. Transcriptomic and metabolomic pathway analyses identified both shared and individual metabolic pathways. Each of the E2, PPT, and DPN treatments downregulated lipid metabolism involving steroid hormone biosynthesis. E2 and PPT suppressed carbohydrate metabolism, including ascorbate and aldarate metabolism; pentose/glucuronate interconversions that are parallel to glycolysis; metabolism of cofactors and vitamins; and, xenobiotics biodegradation and metabolism. Furthermore, E2 enhanced starch and sucrose metabolism and conversion to glucose and G6P, whereas decreased amino acid metabolism involving glycine, serine, and threonine, which are growth-stimulating amino acids [50]. PPT downregulated carbohydrate metabolism involving butanoate metabolism and lipid metabolism, primary involving bile acid biosynthesis. These metabolic changes by E2 and ER agonists provided lower amounts of energy and fewer intermediates as building blocks essential for HepG2 cells to proliferate and grow [51,52].

E2 and ER agonists affected the expressions of regulatory genes in glucose and lipid metabolism in HepG2 cells. The switch from glucose production to glucose usage is a unique feature in HCC metabolism. Cancer cells increase glycolysis and pentose phosphate pathway while reducing gluconeogenesis, both of which contribute to cancer cell survival and growth [17]. It is noteworthy that glycolysis accumulates glycolytic intermediates, such as NADPH and ribose-5-phosphate nucleotide synthesis [53,54]. In cancer cells, the activities of enzymes that are involved in glucose catabolism, such as hexokinase, 6PFK, and PK, are increased in HCC [55] to stimulate cell proliferation. Pentose/glucuronate interconversion occurring in the pentose phosphate pathway, a metabolic pathway parallel to glycolysis, not only provides cancer cells an alternative mechanism for glucose oxidation, but also produces ribose-5-phosphate. Ribose-5-phosphate is a key building block of nuclei acids that facilitate rapid cell division and growth in HCC. On the contrary, the activities of key enzymes that are involved in gluconeogenesis, such as PEPCK1 and PGC1α, are suppressed in primary human HCC and during hepatocarcinogenesis in a mouse model leading to reduced gluconeogenesis in HCC [56,57,58]. E2 and ER agonists have opposite effects on glucose and lipid metabolism when compared to cancer cells.

The expression of the genes that are related to glucose and lipid metabolism collectively indicated suppressed glucose usage with elevated fuel level in HepG2 cells by E2 and ER agonists. E2, PPT, and DPN inhibited the expression of *6PFK* and *PK,* which encode two energetically irreversible glycolytic enzymes, but did not inhibit the expression of *COX6B,* encoding an OXPHOS enzyme. This suggests that estrogen suppresses glycolysis and related TCA cycle via ERα and ERβ, without affecting OXPHOS, in order to suppress glucose usage for cell growth. Additionally, enhanced expressions of *GLUT2*, *PYGL*, *PEPCK1*, and *PGC1A* indicated enhanced glucose uptake, glycogen breakdown, and gluconeogenesis in E2- (*GLUT2*, *PYGL*, and *PGC1A*), ERα agonist- (*PEPCK1*), or ERβ agonist- (*PGC1A*) treated HepG2 cells. Our results also showed the upregulated expressions of transcriptional factors *PPARG* and *SREBP1C,* which activates *de novo* lipogenesis, along with a key lipogenic enzyme, *ACC*, when HepG2 cells were treated with E2 and/or ER agonists. Lipid *de novo* synthesis could play either protective roles or oncogenic roles, depending on different types of cancer. Specifically, as cancer cells shift from OXPHOS to glycolysis, intermediate metabolite pyruvate accumulates and drives cells into *de novo* lipogenesis to meet the need of lipid-rich membrane production [59,60]. The activation of PPARG in HCC cells induces apoptosis and cell cycle arrest, but it inhibits cell proliferation and growth [61,62,63,64]. Consequently, PPARG could be a target for HCC prevention and treatment.

Transcriptome functional pathway analysis also revealed upregulated HIF-1 signaling pathway by E2 and DPN treatments. Hypoxia is a prominent characteristic that is closely related to the abnormal energy metabolism seen in tumor cells, due to rapid cell proliferation and inadequate oxygen supply. Unlike healthy cells, the metabolism of most cancer cells is reprogrammed from mitochondrial oxidative phosphorylation to glycolysis and produces lactic acid to generate energy under the hypoxic condition typically present in tumor microenvironment [65]. Although the expression of *HIF1* or *HIF2* was not significantly different, this study revealed that E2 and DPN commonly upregulated hypoxia-induced genes, such as epidermal growth factor receptor (*EGFR*), egl nine homologue 3 (*EGLN3*), and serpin family E member 1 (*SERPINE1*) (Supplemental Tables S1 and S4) [66,67]. HIF-1 signaling pathway plays a dual role in tumor growth [68]. On one hand, HIF-1 expression is elevated in tumor cells and inhibition of HIF-1 expression suppresses cancer development [69,70], indicating that HIF-1 signaling has oncogenic effects. On the other hand, the overexpression, sustained expression, or activation of HIF-1 impairs tumor growth [71,72], but deficiency or inhibition of HIF-1 promotes tumor growth and survival [73,74,75,76]. Therefore, HIF-1 signaling increases oxygen supply for glycolysis via angiogenesis, provides energy for tumor survival, but it also induces cell cycle arrest and apoptosis, especially when oxygen supply is limited in malignant cancers, such as HCC [68].

### 4.4. Effects of E2 and ER Agonists on Metabolite Regulation in HepG2 Cells

Relatively similar metabolic profiles between E2 and ERα selective agonist PPT, as compared to ERβ selective agonist DPN, were observed (Figure 4 and Figure 5; Supplemental Figure S3). Both ERα and ERβ protein expression were detected in liver HepG2 cells, with ERα expression being more abundant than ERβ expression (Supplemental Figure S1), consistent with the literature [13,77]. Additionally, E2 binding affinity is four-fold higher for ERα than for ERβ [77,78]. Therefore, E2 mainly acts via ERα and it provokes metabolic effects that are relatively similar to ERα selective agonist PPT, in contrast to the very distinct metabolic effects that are produced by ERβ selective agonist DPN.

A high level of ATP or a low ADP/ATP ratio is essential for proliferating and growing cancer cells. Our targeted metabolomics showed that E2 treatment significantly increased, while PPT and DPN treatments had a trend to increase the ADP/ATP ratio as compared to the control treatment (Figure 5). These results suggest dampened fuel utilization, consistent with the suppressed expression of glycolytic genes that contributes to lowered ATP production in HepG2 cells by E2 and ER agonists, consequently leading to suppressed cancer cell growth. Our targeted metabolomics data also showed that E2 and ER agonists significantly affected major metabolites in the metabolism of amino acids, carbohydrates, lipids, cofactors and vitamins, and nucleotides (Table 3; Supplemental Tables S6–S8). Specifically, levels of glyceraldehyde, an intermediate from the breakdown of fructose-1-phosphate that connects fructose metabolism to glycolysis and gluconeogenesis [79], were increased by E2 and ER agonists. The E2 and ER agonists also increased the levels of a nicotinic acid metabolite 6-hydroxynicotinate and 2-hydroxybutyric acid derived from ketobutyric acid. 6-Hydroxynicotinate is a metabolomics biomarker for oral cancer [80]; however, its presence in liver cancer cells has not been extensively reported. 2-Hydroxybutyric acid is produced by the catabolism of amino acid L-threonine and glutathione synthesis in mammalian hepatic cells, whose levels arise due to enhanced lipid oxidation and oxidative stress, as well as impaired glucose regulation [81]. The rate of hepatic glutathione synthesis dramatically increases under oxidative stress or detoxification of xenobiotics in the liver. Therefore, increased 2-hydroxybutyrate in E2- and ER agonist-treated cells may indicate increased oxidative stress. FAD is a redox-active coenzyme associated with different types of proteins and it involves several important enzymatic reactions in metabolism, and it is utilized in energetically difficult oxidation reactions. FAD-dependent proteins function in metabolic pathways, including amino acid catabolism, fatty acid β-oxidation, and biosynthesis of nucleotides and cofactors. One well-known reaction is part of the TCA cycle, in which FAD is required for succinate dehydrogenase of the electron transport chain to catalyze succinate oxidation [82]. In our analysis, the increased FAD production in all three treatment groups may indicate that, due to the restricted glycolysis pathway during the treatments, the energy supplies for these cells have to go through a slightly more active TCA cycle to maintain the energy homeostasis.

Higher levels of G6P, an immediate substrate for both glycolysis and pentose phosphate pathways, were seen in all three treatment groups as compared to the control group. G6P and other measured metabolic intermediates are not final products, but they are able to directly and indirectly impact multiple alternative pathways, including glycolysis, pentose phosphate pathway, TCA cycle, fatty acid biosynthesis, and oxidation, via allosteric regulation of enzymes. The findings of this study clearly indicated significant differences in the levels of key metabolic intermediates between groups that were treated with estrogen or an estrogen receptor agonist and the control group. Several genes and intermediate metabolites were measured, but many other genes and metabolites, along with these gene product proteins, are awaiting further investigation.

### 4.5. Multi-Omics Integration Analysis Revealed Other Pathway Impacts in Addition to Glycolysis

Multi-omics integration analyses confirmed the effects of E2 and its receptor agonists on metabolic and signaling pathways in HepG2 cells. First, we identified eight metabolic enzymes in E2-treated HepG2 cells (Figure 6A) that are key predictors of cancer progression by systematically evaluating the changes of transcriptome and metabolome data and digging into the existing Ingenuity Knowledge Base of IPA. For example, high expression of phosphoglycerate kinase promotes HCC tumorigenesis [83] and phosphoglycerate mutase promotes oral cancer squamous cell migration [84]. Expression of phosphoglycerate kinase has been reported to correlate with tumorigenesis in various types of cancer cells [83,85,86,87,88,89,90]. The overexpression or administration of phosphoglycerate kinase has been reported to promote liver cancer cell growth [83] while inhibiting cell growth and progression in lung, pancreatic, breast, and colon cancer cell lines [87,88]. Therefore, phosphoglycerate kinase could have both oncogenic and anticancer characteristics, and could potentially be an important target for cancer therapy. We also mapped the interaction between differentially expressed genes and detected metabolites, and summarized core genes and metabolites that were affected by all three treatments. Two identified genes at the center of the gene–metabolite interaction map, *SSTR1* and *C5AR1*, were key hubs intertwining with nine metabolites (Figure 6B). It has been reported that healthy hepatocytes do not express any SSTR subtype, while cirrhotic HCC cells and cultured hepatoma cells express all five SSTRs at both mRNA and protein levels [91]. Additionally, an elevated expression of SSTR1 in tumor cells has been reported in patients diagnosed with advanced-stage HCC [92]. In this study, all of the E2 and ER agonist treatments reduced *SSTR1* expression. Interestingly, none of the specific SSTR agonists tested by Reynaert et al. influenced liver cancer proliferation or apoptosis, and only an SSTR1 agonist reduced the migration of HepG2 cells [91]; this suggesting that the stimulation of SSTR1 may decrease invasiveness of HCC via reducing cell migration. The expression of *C5AR1* is increased in HCC and HCC-derived cell lines. Increased expression of *C5AR1* correlates with tumor stage and tumor cell invasion of liver capsule [93]. The complement system plays tumor promoting roles. The activation of the complement system promotes tumor growth via providing complement mediators and an inflammatory microenvironment [94]. Several complement components have been reported to regulate tumor growth via directly interacting with their corresponding receptors on tumor cells. The interaction of complement C5a and its receptor C5aR1 activates the mitogenic pathway and decreases apoptosis, consequently promoting oncogenesis [95]. However, most of this line of research has focused on the roles of complement in regulating growth of primary tumors. In the current study, the expression of *C5AR1* was downregulated by E2, leading to reduced interaction between C5 and its receptor C5AR1, but it was upregulated by PPT and DPN possibly due to lowered C5 production by HepG2 cells. This possibility and the complicated involvement of C5aR1 signaling pathway in cancer development and metastasis await further investigation. Several metabolites that were reported in the gene–metabolite interaction map were key amino acid metabolites (i.e., serotonin and melatonin in tryptophan metabolism; dopamine, norepinephrine, and epinephrine in tyrosine metabolism; histamine in histidine metabolism; and, gamma-aminobutyric acid in glutamate metabolism), guanine in nucleotide metabolism, and acetylcholine in glycerophospholipid metabolism. This finding suggested that, in addition to glycolysis pathway, the metabolism of amino acids, nucleotides, and glycerophospholipids could be investigated in future studies.

## 5. Conclusions

HCC is one of the most common and deadly cancers worldwide [1,2]. Warburg’s discovery regarding an increased utilization of aerobic glycolysis not only establishes metabolic reprogramming as one of the distinguishing characteristics of cancer cells [96], but also provides a venue for developing anticancer treatments [97]. Additional metabolic alterations in cancer, such as pentose phosphate pathway, amino acid metabolism, and nucleotide metabolism, have also been revealed to contribute to metabolism reprogramming [50]. We investigated the metabolic effects of estrogen and selective nuclear estrogen receptor agonists in HCC cells, and identified metabolic pathways using integrated transcriptome and metabolome analyses. It is noteworthy that estrogen acts on classic nuclear ERs, including ERα and ERβ, as well as membrane-bound ERs, including G protein-coupled ER (GPER, also known as GPR 30) and membrane-associated ERα and ERβ variants, in order to exert metabolic effects in glucose and lipid metabolism in liver cells [10]. The findings collectively indicated elevated intracellular fuel levels, via upregulated conversion from starch and sucrose to glucose, glucose uptake, glycogenolysis, gluconeogenesis, and lipogenesis, with dampened energy utilization, via inhibited glycolysis and its parallel pentose/glucuronate interconversion pathway, but unaffected mitochondrial OXPHOS. This led to impaired ATP levels in HepG2 cells, which serves as a potential mechanism underlying anticancer effects and provides a strategy for HCC treatment. Certain genes and metabolites identified in this study could be targets of future liver cancer treatment.

## Figures and Tables

**Table 1:** Primers for reverse transcription-quantitative PCR.

Gene	Coding Protein	Forward Primers (5′–3′)	Reverse Primers (5′–3′)
*ACC*	acetyl-CoA carboxylase	GCTGCTCGGATCACTAGTGAA	TTCTGCTATCAGTCTGTCCAG
*ACTB*	β actin	AGAGCTACGAGCTGCCTGAC	AGCACTGTGTTGGCGTACAG
*COX6B*	cytochrome c oxidase subunit 6B	CTCAACGTGTTCCTCAAGTC	ATGGAGGACAGAGGAAAGG
*FAS*	fatty acid synthase	GAAACTGCAGGAGCTGTC	CACGGAGTTGAGGCGGAT
*GLUT2*	glucose transporter 2	AGTTAGATGAGGAAGTCAAAGCAA	TAGGCTGTCGGTAGCTGG
*GYS2*	glycogen synthase 2	GCCAGACACCTGACATTAAG	CTCCACTTCATCTTCCACATC
*PEPCK1*	phosphoenolpyruvate carboxykinase	CCAGGCAGTGAGGGAGTTTCT	ACTGTGTCTCTTTGCTCTTGG
*6PFK*	6-phosphofructokinase	CTCACAGGTGCCAACATC	GCCGCAGAAGTCGTTATC
*PGC1A*	PPAR gamma coactivator 1 alpha	GACGACGAAGCAGACAAG	CCAAGGGTAGCTCAGTTTATC
*PK*	pyruvate kinase	TCGTCTTTGCCTCCTTTG	CTCACCTCCAGGATTTCATC
*PPARG*	PPAR gamma	GAAATGACCATGGTTGAC	CCGCTAGTACAAGTCCTTGTA
*PYGL*	glycogen phosphorylase liver form	CCTGTGATGAGGCCATTTAC	GTATCCATAGGCTGCAAGTC
*SREBP1C*	sterol regulatory element-binding transcription factor 1	CTTTGCCCACCCTGGTGAGT	GGTTCTCCTGCTTGAGTTTCTGG

**Table 2:** Significant pathways of HepG2 cells treated with estradiol estradiol (E2), ERα specific agonist PPT, and ERβ specific agonist DPN revealed by transcriptome pathway analysis.

Upregulated Pathways	Treatment Group
HIF-1 signaling pathway	E2 DPN
Complement & coagulation cascades	E2 DPN
Carbohydrate digestion & absorption	E2
Starch & sucrose metabolism	E2
Hematopoietic cell lineage	DPN
**Downregulated Pathways**	**Treatment Group**
Steroid hormone biosynthesis	E2 PPT DPN
Cell cycle	E2 PPT DPN
Progesterone-mediated oocyte maturation	E2 PPT DPN
Oocyte meiosis	E2 PPT DPN
p53 Signaling pathway	E2 PPT DPN
Ascorbate and aldarate metabolism	E2 PPT
Pentose and glucuronate interconversions	E2 PPT
Retinol metabolism	E2 PPT
Metabolism of xenobiotics by cytochrome P450	E2 PPT
Drug metabolism	E2 PPT
Chemical carcinogenesis	E2 PPT
Porphyrin and chlorophyll metabolism	E2 PPT
Fanconi anemia pathway	PPT DPN
Glycine, serine & threonine metabolism	E2
Butanoate metabolism	PPT
Primary bile acid biosynthesis	PPT
Complement and coagulation cascades	PPT
PPAR signaling pathway	PPT
DNA replication	DPN
Base excision repair	DPN
Nucleotide excision repair	DPN
Mismatch repair	DPN
Homologous recombination	DPN
Bladder cancer	DPN
HTLV-I infection	DPN

**Table 3:** Significant metabolic pathways of HepG2 cells treated with estradiol E2, ERα specific agonist PPT, and ERβ specific agonist DPN revealed by metabolomic pathway analysis.

Metabolism	Pathways	Treatment Group
Amino acid	Tyrosine metabolism	E2 PPT DPN
	Tryptophan metabolism	E2 PPT DPN
	Histidine metabolism	E2 PPT DPN
	Glycine, serine and threonine metabolism	E2 PPT DPN
	Arginine and proline metabolism	E2 PPT DPN
	Taurine and hypotaurine metabolism	E2 PPT DPN
	Cysteine and methionine metabolism	PPT DPN
	beta-Alanine metabolism	PPT DPN
	Arginine biosynthesis	PPT DPN
	Alanine, aspartate and glutamate metabolism	PPT DPN
	Glutathione metabolism	PPT DPN
Carbohydrate	Amino sugar and nucleotide sugar metabolism	E2 PPT DPN
	Pyruvate metabolism	E2 PPT DPN
	Citrate cycle (TCA cycle)	E2 PPT DPN
	Glycolysis and gluconeogenesis	E2 PPT DPN
	Glyoxylate and dicarboxylate metabolism	PPT DPN
Lipid	Glycerophospholipid metabolism	PPT DPN
Cofactors and vitamins	One carbon pool by folate	E2 DPN
	Vitamin B6 metabolism	PPT DPN
	Nicotinate and nicotinamide metabolism	PPT DPN
	Biotin metabolism	DPN
Nucleotide	Pyrimidine metabolism	PPT DPN
Biosynthesis of other metabolites	Caffeine metabolism	PPT DPN
Genetic information translation processing	Aminoacyl-tRNA biosynthesis	PPT DPN

## Data Availability

RNA sequencing data are available at Gene Expression Omnibus (accession number GSE112983).

## Supplementary Materials

The following are available online at https://www.mdpi.com/2073-4409/10/2/455/s1, Supplemental Tables S1–S5. Effects of estradiol, estrogen receptor ERα agonist PPT, and estrogen receptor ERβ agonist DPN on gene expression detected via RNA sequencing and KEGG pathway analysis. Table S1: Upregulated genes and pathways by estradiol comparing to control treatment. Table S2: Downregulated genes and pathways by estradiol comparing to control treatment. Table S3: Downregulated genes and pathways by ERα agonist PPT comparing to control treatment. Table S4: Upregulated genes and pathways by ERβ agonist DPN comparing to control treatment. Table S5: Downregulated genes and pathways by ERβ agonist DPN comparing to control treatment. Supplemental Tables S6–S8. Effects of estradiol, estrogen receptor ERα agonist PPT, and estrogen receptor ERβ agonist DPN on metabolites detected via metabolite profiling and KEGG pathway analysis. Table S6: Altered metabolites and pathways by estradiol comparing to control treatment. Table S7: Altered metabolites and pathways by ERα agonist PPT comparing to control treatment. Table S8: Altered metabolites and pathways by ERβ agonist DPN comparing to control treatment. Supplemental Figure S1. Western blot analysis detected protein expression of ERα (66 kDa), ERβ (56 kDa), and a housekeeping protein β-actin (45 kDa) in HepG2 cells used in this study. The gel blot image shows protein bands and a protein ladder as the molecular size marker. Supplemental Figure S2. Representative images of HepG2 cells treated with 2-deoxy-D-glucose, oxamate, and oligomycin. HepG2 cells treated with vehicle DMSO (Control), 2-deoxy-D-glucose (5 and 10 mM), oxamate (5, 10 and 50 mM), or oligomycin (0.1, 0.5 and 1.0 µg/mL) were evaluated using light microscopy (10 × magnification). Bars = 50 μm. Supplemental Figure S3. Heatmap presentation of metabolic profiles comparing relative concentrations of detected 174 metabolites from HepG2 cells treated with control, estradiol (E2), ERα specific agonist PPT, and ERβ specific agonist DPN. Each column represents one biological replicate, and each row represents one targeted metabolite detected. Supplemental Figure S4. Gene–metabolite interaction maps. Gene–metabolite interaction maps showing the effect of estradiol (S4A), ERα specific agonist PPT (S4B), and ERβ specific agonist DPN (S4C) on the transcriptome and metabolome of HepG2 cells using significantly differentially expressed genes identified by RNA-Seq analysis and their significantly altered metabolites detected by targeted metabolomics. Each square note represents one metabolite, and each round note represents one gene. The size of the nodes is proportional to their degree values, signifying the number of connections it has to other nodes. Nodes with higher node degree act as hubs in a network. The color of nodes reflects their betweenness centrality values, the number of shortest paths, or interactions going through the node. Figure S4A: Gene–metabolite interaction maps showing the effect of estradiol. Figure S4B: Gene–metabolite interaction maps showing the effect of ERα agonist PPT. Figure S4C: Gene–metabolite interaction maps showing the effect of ERβ agonist DPN.
