# Supplementary material for: A Multi-Omics Study Revealing the Metabolic Effects of Estrogen in Liver Cancer Cells HepG2
Source: Cells. 2021 Feb 20;10(2):455. doi: 10.3390/cells10020455 (PMC7924215; doi:10.3390/cells10020455)

SUPPLEMENTAL INFORMATION

A Multi-Omics Study Revealing the Metabolic Effects of Estrogen in Liver Cancer Cells HepG2

Minqian Shen, Mengyang Xu, Fanyi Zhong, McKenzie C. Crist, Anjali B. Prior, Kundi Yang, Danielle M. Allaire, Fouad Choueiry, Jiangjiang Zhu, Haifei Shi

Supplemental Tables

Supplemental Tables 1-5. Effects of estradiol, ERα agonist PPT, and ERβ agonist DPN on gene expression detected via RNA sequencing and KEGG pathway analysis.

Supplemental Table 1. Upregulated genes and pathways by estradiol comparing to control treatment.

Genome functional pathways	Group	P Value	Adjusted P Value	# Total Hits	# Hits	Associated Genes
Carbohydrate digestion and absorption	Group 3	3.7E-8	1.1E-7	15	13	[AMY1A, AMY1B, AMY1C, AMY2A, AMY2B, ATP1A1, ATP1B2, FXYD2, G6PC, HK1, MGAM, MGAM2, SLC2A5]
Starch and sucrose metabolism					11	[AMY1A, AMY1B, AMY1C, AMY2A, AMY2B, G6PC, GBE1, GYS1, HK1, MGAM, MGAM2]
Complement and coagulation cascades	Group 2	3.2E-6	6.5E-6	14	14	[C3, C4A, C4B, C7, C8B, F11, F5, ITGB2, KLKB1, PLG, SERPINE1, SERPING1, VTN, VWF]
HIF-1 signaling pathway	Group 1	5.9E-5	5.9E-5	14	14	[CAMK2A, EDN1, EGFR, EGLN1, EGLN3, ENO2, ENO3, EPO, HK1, IGF1R, PDK1, PFKFB3, SERPINE1, VEGFA]

Supplemental Table 2. Downregulated genes and pathways by estradiol comparing to control treatment.

Genome functional pathways	Group	P Value	Adjusted P Value	# Total Hits	# Hits	Associated Genes
Ascorbate and aldarate metabolism	Group 4	2.7E-10	1.0E-9	23	13	[UGT1A1, UGT1A10, UGT1A3, UGT1A4, UGT1A5, UGT1A6, UGT1A7, UGT1A8, UGT1A9, UGT2B10, UGT2B11, UGT2B28, UGT2B4]
Pentose and glucuronate interconversions					14	[AKR1B10, UGT1A1, UGT1A10, UGT1A3, UGT1A4, UGT1A5, UGT1A6, UGT1A7, UGT1A8, UGT1A9, UGT2B10, UGT2B11, UGT2B28, UGT2B4]
Retinol metabolism					17	[ADH4, ADH6, ALDH1A1, RDH5, UGT1A1, UGT1A10, UGT1A3, UGT1A4, UGT1A5, UGT1A6, UGT1A7, UGT1A8, UGT1A9, UGT2B10, UGT2B11, UGT2B28, UGT2B4]
Metabolism of xenobiotics by cytochrome P450					17	[ADH4, ADH6, GSTA1, GSTA2, UGT1A1, UGT1A10, UGT1A3, UGT1A4, UGT1A5, UGT1A6, UGT1A7, UGT1A8, UGT1A9, UGT2B10, UGT2B11, UGT2B28, UGT2B4]
Drug metabolism					14	[TK1, UGT1A1, UGT1A10, UGT1A3, UGT1A4, UGT1A5, UGT1A6, UGT1A7, UGT1A8, UGT1A9, UGT2B10, UGT2B11, UGT2B28, UGT2B4]
Steroid hormone biosynthesis					15	[AKR1D1, SULT1E1, UGT1A1, UGT1A10, UGT1A3, UGT1A4, UGT1A5, UGT1A6, UGT1A7, UGT1A8, UGT1A9, UGT2B10, UGT2B11, UGT2B28, UGT2B4]
Chemical carcinogenesis					17	[ADH4, ADH6, GSTA1, GSTA2, UGT1A1, UGT1A10, UGT1A3, UGT1A4, UGT1A5, UGT1A6, UGT1A7, UGT1A8, UGT1A9, UGT2B10, UGT2B11, UGT2B28, UGT2B4]
Porphyrin and chlorophyll metabolism					13	[UGT1A1, UGT1A10, UGT1A3, UGT1A4, UGT1A5, UGT1A6, UGT1A7, UGT1A8, UGT1A9, UGT2B10, UGT2B11, UGT2B28, UGT2B4]
Cell cycle	Group3	1.7E-4	3.4E-4	14	12	[BUB1, CCNA2, CCNB1, CCNB2, CDC20, CDC25A, CDC25C, CDK1, CDKN2C, PLK1, PTTG1, TTK]
Progesterone-mediated oocyte maturation					9	[BUB1, CCNA2, CCNB1, CCNB2, CDC25A, CDC25C, CDK1, PIK3R3, PLK1]
Oocyte meiosis					10	[AURKA, BUB1, CCNB1, CCNB2, CDC20, CDC25C, CDK1, FBXO5, PLK1, PTTG1]
Glycine, serine and threonine metabolism	Group2	2.1E-5	6.5E-5	7	7	[BPGM, CTH, DAO, GLDC, PHGDH, PSAT1, SHMT2]
p53 Signaling pathway	Group1	7.4E-4	7.4E-4	7	7	[CCNB1, CCNB2, CDK1, GTSE1, RRM2, SESN2, SESN3]

Supplemental Table 3. Downregulated genes and pathways by PPT comparing to control treatment.

Genome functional pathways	Group	P Value	Adjusted P Value	# Total Hits	# Hits	Associated Genes
Steroid hormone biosynthesis	Group 8	1.3E-6	9.7E-6	18	15	[AKR1D1, CYP17A1, CYP7A1, SULT1E1, UGT1A1, UGT1A10, UGT1A3, UGT1A4, UGT1A5, UGT1A6, UGT1A7, UGT1A8, UGT1A9, UGT2B10, UGT2B11]
Ascorbate and aldarate metabolism					11	[UGT1A1, UGT1A10, UGT1A3, UGT1A4, UGT1A5, UGT1A6, UGT1A7, UGT1A8, UGT1A9, UGT2B10, UGT2B11]
Pentose and glucuronate interconversions					11	[UGT1A1, UGT1A10, UGT1A3, UGT1A4, UGT1A5, UGT1A6, UGT1A7, UGT1A8, UGT1A9, UGT2B10, UGT2B11]
Drug metabolism					12	[TK1, UGT1A1, UGT1A10, UGT1A3, UGT1A4, UGT1A5, UGT1A6, UGT1A7, UGT1A8, UGT1A9, UGT2B10, UGT2B11]
Porphyrin and chlorophyll metabolism					11	[UGT1A1, UGT1A10, UGT1A3, UGT1A4, UGT1A5, UGT1A6, UGT1A7, UGT1A8, UGT1A9, UGT2B10, UGT2B11]
Retinol metabolism					12	[RDH5, UGT1A1, UGT1A10, UGT1A3, UGT1A4, UGT1A5, UGT1A6, UGT1A7, UGT1A8, UGT1A9, UGT2B10, UGT2B11]
Metabolism of xenobiotics by cytochrome P450					12	[GSTM2, UGT1A1, UGT1A10, UGT1A3, UGT1A4, UGT1A5, UGT1A6, UGT1A7, UGT1A8, UGT1A9, UGT2B10, UGT2B11]
Chemical carcinogenesis					12	[GSTM2, UGT1A1, UGT1A10, UGT1A3, UGT1A4, UGT1A5, UGT1A6, UGT1A7, UGT1A8, UGT1A9, UGT2B10, UGT2B11]
Cell cycle	Group7	3.8E-11	3.0E-10	25	22	[BUB1, BUB1B, CCNA2, CCNB1, CCNB2, CDC20, CDC25C, CDC45, CDK1, CDKN2C, CHEK1, E2F1, MAD2L1, MCM2, MCM5, ORC1, PCNA, PLK1, PTTG1, SKP2, TGFB3, TTK]
Oocyte meiosis					12	[AURKA, BUB1, CCNB1, CCNB2, CDC20, CDC25C, CDK1, FBXO43, MAD2L1, PLK1, PTTG1, SGO1]
Progesterone-mediated oocyte maturation					8	[BUB1, CCNA2, CCNB1, CCNB2, CDC25C, CDK1, MAD2L1, PLK1]
Butanoate metabolism	Group6	3.1E-3	3.1E-3	4	4	[ACSM2A, ACSM2B, ACSM3, HMGCS2]
Primary bile acid biosynthesis	Group5	4.4E-4	1.7E-3	4	4	[AKR1D1, BAAT, CYP7A1, CYP8B1]
Complement and coagulation cascades	Group4	8.6E-6	5.1E-5	10	10	[C6, C7, C8A, C8B, CFI, CPB2, F13B, MBL2, SERPINA5, SERPIND1]
p53 signaling pathway	Group3	1.9E-5	9.6E-5	9	9	[CCNB1, CCNB2, CDK1, CHEK1, DDB2, GTSE1, RRM2, SESN3, TP73]

Fanconi anemia pathway	Group2	1.2E-3	2.5E-3	6	6	[BLM, BRIP1, FANCB, FANCD2, RAD51, RMI2]
PPAR signaling pathway	Group1	1.0E-3	3.1E-3	7	7	[ACSL6, ANGPTL4, CYP7A1, CYP8B1, HMGCS2, SORBS1, UCP1]

Supplemental Table 4. Upregulated genes and pathways by DPN comparing to control treatment.

Genome functional pathways	Group	P Value	Adjusted P Value	# Total Hits	# Hits	Associated Genes
Hematopoietic cell lineage	Group 3	1.9E-3	3.9E-3	6	6	[CD22, CD9, IL11, IL6R, ITGA2, ITGA3]
Complement and coagulation cascades	Group 2	6.8E-4	2.0E-3	6	6	[C5AR1, C8G, ITGAX, PROCR, SERPINE1, SERPING1]
HIF-1 signaling pathway	Group 1	2.4E-3	2.4E-3	6	6	[EGFR, EGLN3, IL6R, NOS3, SERPINE1, TIMP1]

Supplemental Table 5. Downregulated genes and pathways by DPN comparing to control treatment.

Genome functional pathways	Group	Group P Value	Adjusted P Value	# Total Hits	# Hits	Associated Genes
DNA replication	Group 7	2.9E-6	1.7E-5	9	6	[LIG1, MCM2, MCM5, MCM7, POLD1, POLE]
Base excision repair					4	[LIG1, NEIL3, POLD1, POLE]
Nucleotide excision repair					4	[DDB2, LIG1, POLD1, POLE]
Mismatch repair					3	[EXO1, LIG1, POLD1]
Cell cycle	Group 6	9.1E-17	6.3E-16	26	21	[BUB1, BUB1B, CCNA2, CCNB1, CCNB2, CDC20, CDC25B, CDC25C, CDC45, CDK1, CDKN2C, E2F1, ESPL1, MAD2L1, MCM2, MCM5, MCM7, ORC1, PLK1, PTTG1, TTK]
Oocyte meiosis					14	[AURKA, BUB1, CCNB1, CCNB2, CDC20, CDC25C, CDK1, ESPL1, FBXO43, FBXO5, MAD2L1, PLK1, PTTG1, SGO1]
Progesterone-mediated oocyte maturation					9	[BUB1, CCNA2, CCNB1, CCNB2, CDC25B, CDC25C, CDK1, MAD2L1, PLK1]
Homologous recombination	Group 5	8.2E-5	3.2E-4	7	5	[BLM, BRIP1, POLD1, RAD51, RAD54L]
Fanconi anemia pathway					5	[BLM, BRIP1, FANCD2, RAD51, RMI2]
p53 Signaling pathway	Group 4	2.8E-5	1.4E-4	7	7	[CCNB1, CCNB2, CDK1, DDB2, GTSE1, RRM2, TP73]
Bladder cancer	Group 3	1.6E-2	1.6E-2	3	3	[E2F1, RPS6KA5, UPK3A]
HTLV-I infection	Group 2	1.1E-4	3.3E-4	12	12	[BUB1B, CCNB2, CDC20, CDKN2C, E2F1, IL15, MAD2L1, MYBL1, MYBL2, POLD1, POLE, PTTG1]
Steroid hormone biosynthesis	Group 1	6.7E-3	1.3E-2	4	4	[AKR1D1, CYP17A1, HSD11B2, SULT1E1]

Supplemental Tables 6-8. Effects of estradiol, ERα specific agonist PPT, and ERβ specific agonist DPN on metabolites detected via metabolite profiling and KEGG pathway analysis.

Supplemental Table 6. Altered metabolites and pathways by estradiol comparing to control treatment.

Metabolic pathways	Total	Hits	P Value	Adjusted P value	FDR	Impact
Tryptophan metabolism	41	8	8.09E-05	3.56E-03	3.05E-03	0.3450
Taurine and hypotaurine metabolism	8	3	1.91E-04	8.21E-03	3.05E-03	0.7140
Histidine metabolism	16	2	3.46E-04	1.42E-02	3.05E-03	0.3110
Tyrosine metabolism	42	10	3.47E-04	1.42E-02	3.05E-03	0.3460
Purine metabolism	65	5	5.52E-04	2.15E-02	3.70E-03	0.0968
Amino sugar and nucleotide sugar metabolism	37	5	6.76E-04	2.57E-02	3.70E-03	0.1160
One carbon pool by folate	9	1	7.56E-04	2.80E-02	3.70E-03	0.7920
Glycine, serine and threonine metabolism	33	9	8.80E-04	3.08E-02	3.87E-03	0.1290
Pyruvate metabolism	22	4	1.39E-03	4.73E-02	4.55E-03	0.2380
Citrate cycle (TCA cycle)	20	4	1.39E-03	4.73E-02	4.55E-03	0.1200
Arginine and proline metabolism	38	7	1.41E-03	4.73E-02	4.55E-03	0.1700
Glycolysis / Gluconeogenesis	26	2	1.45E-03	4.73E-02	4.55E-03	0.2060

Supplemental Table 7. Altered metabolites and pathways by PPT comparing to control treatment.

Metabolic pathways	Total	Hits	P Value	Adjusted P value	FDR	Impact
Tyrosine metabolism	42	10	1.04E-11	4.57E-10	4.57E-10	0.3460
Glycerophospholipid metabolism	36	4	2.77E-08	1.19E-06	4.03E-07	0.0872
Taurine and hypotaurine metabolism	8	3	3.66E-08	1.50E-06	4.03E-07	0.7143
Amino sugar and nucleotide sugar metabolism	37	5	2.42E-07	9.69E-06	2.13E-06	0.1159
Vitamin B6 metabolism	9	3	7.61E-07	2.97E-05	5.58E-06	0.6471
Pyrimidine metabolism	39	6	1.81E-06	6.89E-05	1.14E-05	0.2146
Tryptophan metabolism	41	8	1.03E-05	3.81E-04	3.74E-05	0.3448
Cysteine and methionine metabolism	33	4	1.07E-05	3.81E-04	3.74E-05	0.3272
Purine metabolism	65	5	1.07E-05	3.81E-04	3.74E-05	0.0968
Pyruvate metabolism	22	4	1.10E-05	3.81E-04	3.74E-05	0.2379
Citrate cycle (TCA cycle)	20	4	1.10E-05	3.81E-04	3.74E-05	0.1203
Histidine metabolism	16	2	1.89E-05	5.66E-04	5.54E-05	0.3115
Nicotinate and nicotinamide metabolism	15	3	2.21E-05	6.41E-04	6.08E-05	0.5671
Glycine, serine and threonine metabolism	33	9	4.39E-05	1.19E-03	1.07E-04	0.1288
Glycolysis / Gluconeogenesis	26	2	5.65E-05	1.47E-03	1.27E-04	0.2059
Glutathione metabolism	28	5	6.40E-05	1.53E-03	1.30E-04	0.0872
Arginine and proline metabolism	38	7	6.50E-05	1.53E-03	1.30E-04	0.1701
Aminoacyl-tRNA biosynthesis	48	2	9.00E-05	1.98E-03	1.72E-04	0.0556
beta-Alanine metabolism	21	3	9.43E-05	1.98E-03	1.73E-04	0.1045
Glyoxylate and dicarboxylate metabolism	32	4	2.30E-04	4.38E-03	3.90E-04	0.2990
Alanine, aspartate and glutamate metabolism	28	4	3.65E-04	6.37E-03	5.56E-04	0.0889
Pentose and glucuronate interconversions	18	1	3.66E-04	6.37E-03	5.56E-04	0.0781
Starch and sucrose metabolism	18	2	6.68E-04	1.00E-02	9.80E-04	0.4304
Arginine biosynthesis	14	3	7.79E-04	1.09E-02	1.11E-03	0.2893
Caffeine metabolism	10	2	1.81E-03	2.17E-02	2.41E-03	0.6923

Supplemental Table 8. Altered metabolites and pathways by DPN comparing to control treatment.

Metabolic pathways	Total	Hits	P Value	Adjusted P value	FDR	Impact
Tyrosine metabolism	42	10	2.75E-11	1.18E-09	6.06E-10	0.3460
Glycerophospholipid metabolism	36	4	1.59E-10	6.69E-09	2.34E-09	0.0872
Taurine and hypotaurine metabolism	8	3	2.97E-10	1.22E-08	3.27E-09	0.7143
Tryptophan metabolism	41	8	5.50E-09	2.20E-07	4.56E-08	0.3448
Amino sugar and nucleotide sugar metabolism	37	5	6.22E-09	2.43E-07	4.56E-08	0.1159
Purine metabolism	65	5	1.06E-08	4.04E-07	6.56E-08	0.0968
Vitamin B6 metabolism	9	3	1.19E-08	4.41E-07	6.56E-08	0.6471
Glutathione metabolism	28	5	2.04E-08	7.34E-07	9.37E-08	0.0872
beta-Alanine metabolism	21	3	2.13E-08	7.45E-07	9.37E-08	0.1045
Cysteine and methionine metabolism	33	4	2.83E-08	9.61E-07	1.13E-07	0.3272
Arginine and proline metabolism	38	7	8.17E-08	2.61E-06	2.63E-07	0.1701
Pyrimidine metabolism	39	6	9.76E-08	2.93E-06	2.81E-07	0.2146
Aminoacyl-tRNA biosynthesis	48	2	1.02E-07	2.96E-06	2.81E-07	0.0556
Glycolysis / Gluconeogenesis	26	2	1.87E-07	5.25E-06	4.85E-07	0.2059
Glycine, serine and threonine metabolism	33	9	2.11E-07	5.69E-06	5.15E-07	0.1288
Pyruvate metabolism	22	4	3.62E-07	9.40E-06	7.96E-07	0.2379
Citrate cycle (TCA cycle)	20	4	3.62E-07	9.40E-06	7.96E-07	0.1203
Nicotinate and nicotinamide metabolism	15	3	5.11E-07	1.23E-05	1.07E-06	0.5671
Arginine biosynthesis	14	3	5.61E-07	1.29E-05	1.12E-06	0.2893
Starch and sucrose metabolism	18	2	8.88E-07	1.87E-05	1.63E-06	0.4304
Alanine, aspartate and glutamate metabolism	28	4	1.89E-06	3.59E-05	3.20E-06	0.0889
Histidine metabolism	16	2	2.37E-06	4.18E-05	3.72E-06	0.3115
Glyoxylate and dicarboxylate metabolism	32	4	3.92E-06	5.90E-05	5.75E-06	0.2990
Caffeine metabolism	10	2	6.22E-06	8.70E-05	8.82E-06	0.6923
Pentose and glucuronate interconversions	18	1	7.58E-06	9.86E-05	1.04E-05	0.0781
One carbon pool by folate	9	1	1.68E-05	1.70E-04	2.06E-05	0.7921
Inositol phosphate metabolism	30	1	7.15E-05	5.01E-04	8.07E-05	0.1294
Biotin metabolism	10	1	2.17E-04	8.67E-04	2.32E-04	0.2000

Supplemental Figures

Supplemental Figure S1. Western blot analysis detected protein expression of ERα (66 kDa), ERβ (56 kDa), and a housekeeping protein β-actin (45 kDa) in HepG2 cells used in this study. The gel blot image shows protein bands and a protein ladder as the molecular size marker.

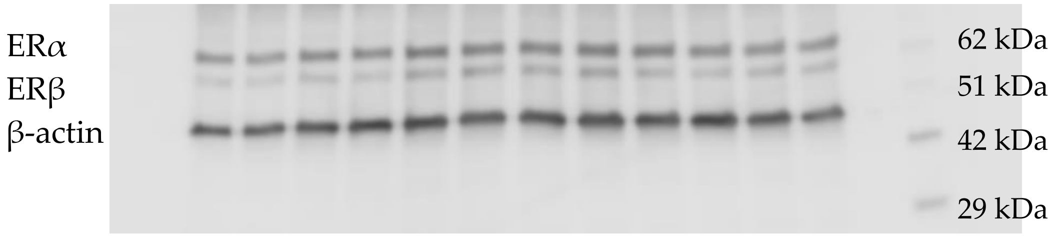

Supplemental Figure S2.

Representative images of HepG2 cells treated with 2-deoxy-D-glucose, oxamate, and oligomycin. HepG2 cells treated with vehicle DMSO (Control), 2-deoxy-D-glucose (5 and 10 mM), oxamate (5, 10, and 50 mM), or oligomycin (0.1, 0.5, and 1.0 µg/ml) were evaluated using light microscopy (10 × magnification). Bars = 50 µm.

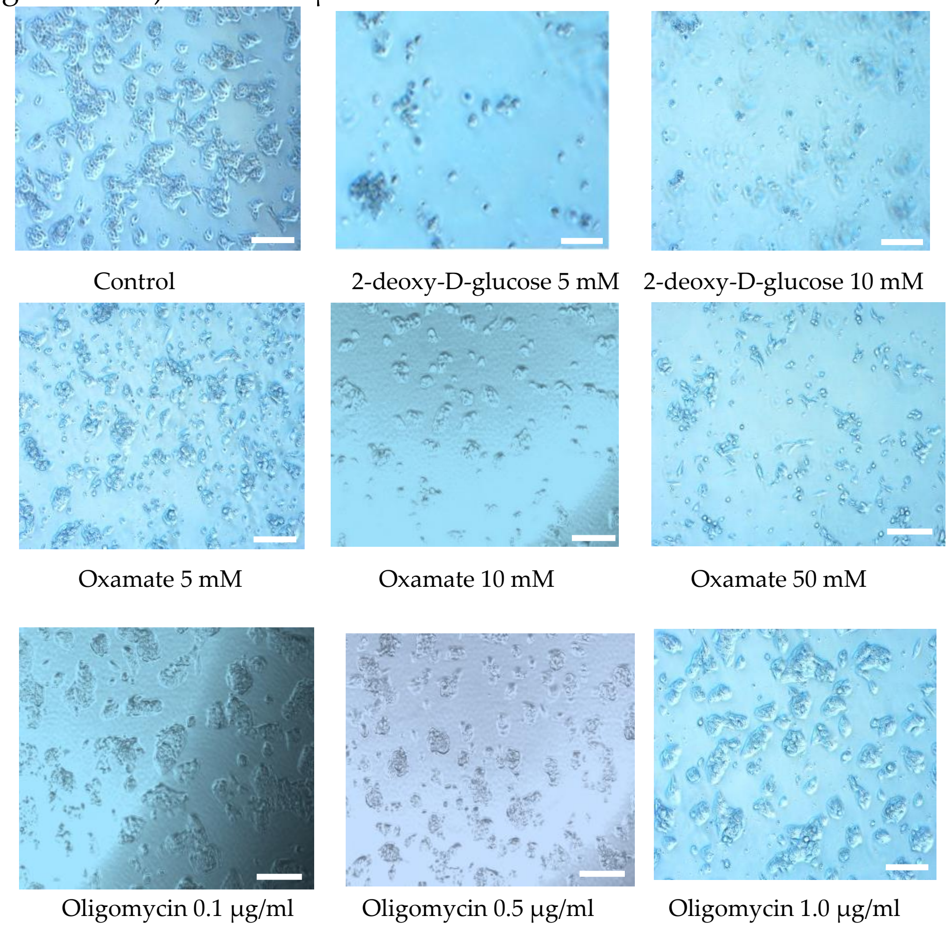

Supplemental Figure S3. Heatmap presentation of metabolic profiles comparing relative concentrations of detected 174 metabolites from HepG2 cells treated with control, estradiol (E2), ERα specific agonist PPT, and ERβ specific agonist DPN. Each column represents one biological replicate, and each row represents one targeted metabolite detected.

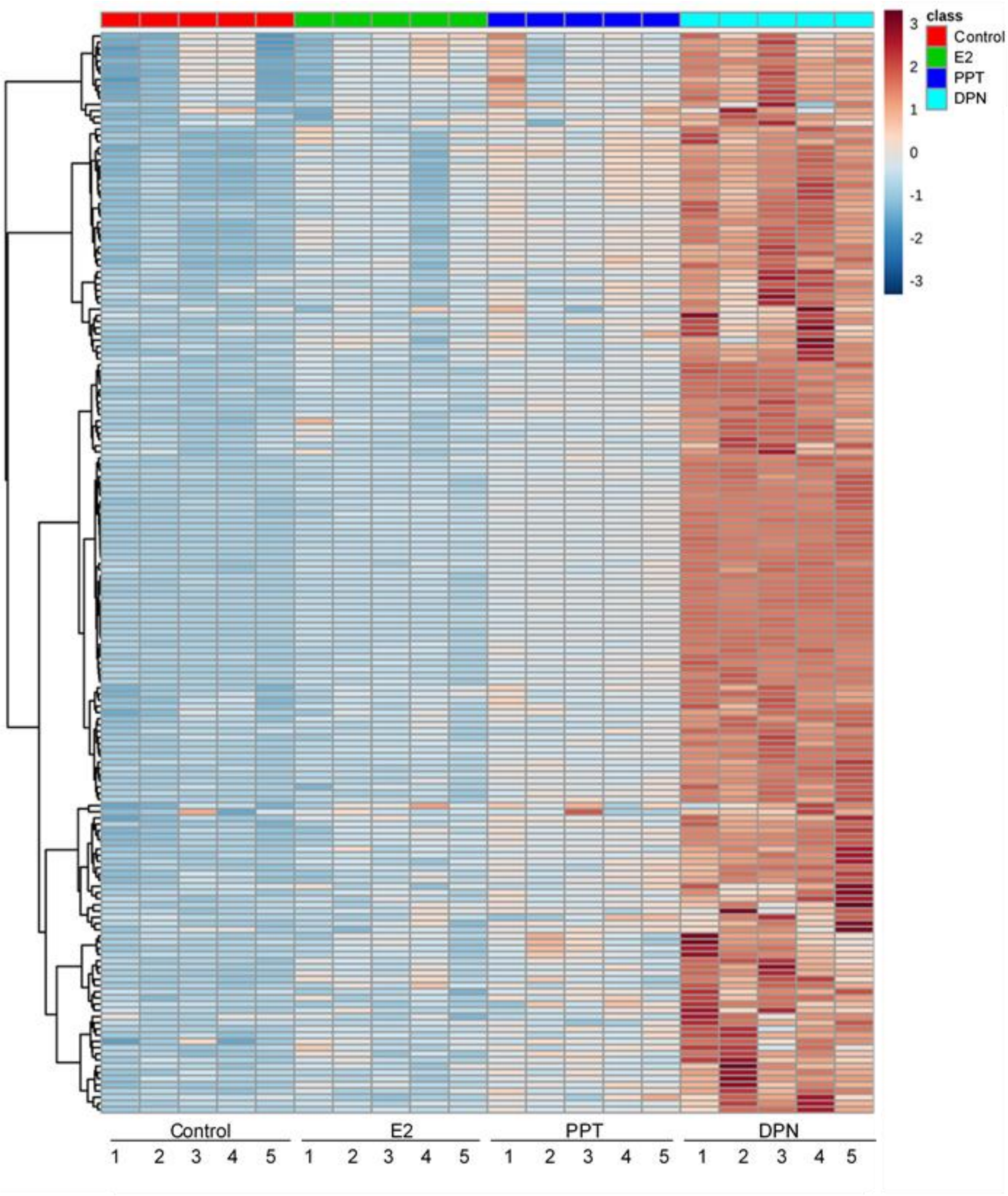

Supplemental Figure 4. Gene-metabolite interaction maps.

Gene-metabolite interaction maps showing the effect of estradiol (S4A), ERα specific agonist PPT (S4B), and ERβ specific agonist DPN (S4C) on the transcriptome and metabolome of HepG2 cells using significantly differentially expressed genes identified by RNA-Seq analysis and their significantly altered metabolites detected by targeted metabolomics. Each square note represents one metabolite, and each round note represents one gene. The size of the nodes is proportional to their degree values, signifying the number of connections it has to other nodes. Nodes with higher node degree act as hubs in a network. The color of nodes reflects their betweenness centrality values, the number of shortest paths, or interactions going through the node.

Supplemental Figure 4A. Gene-metabolite interaction maps showing the effect of estradiol.

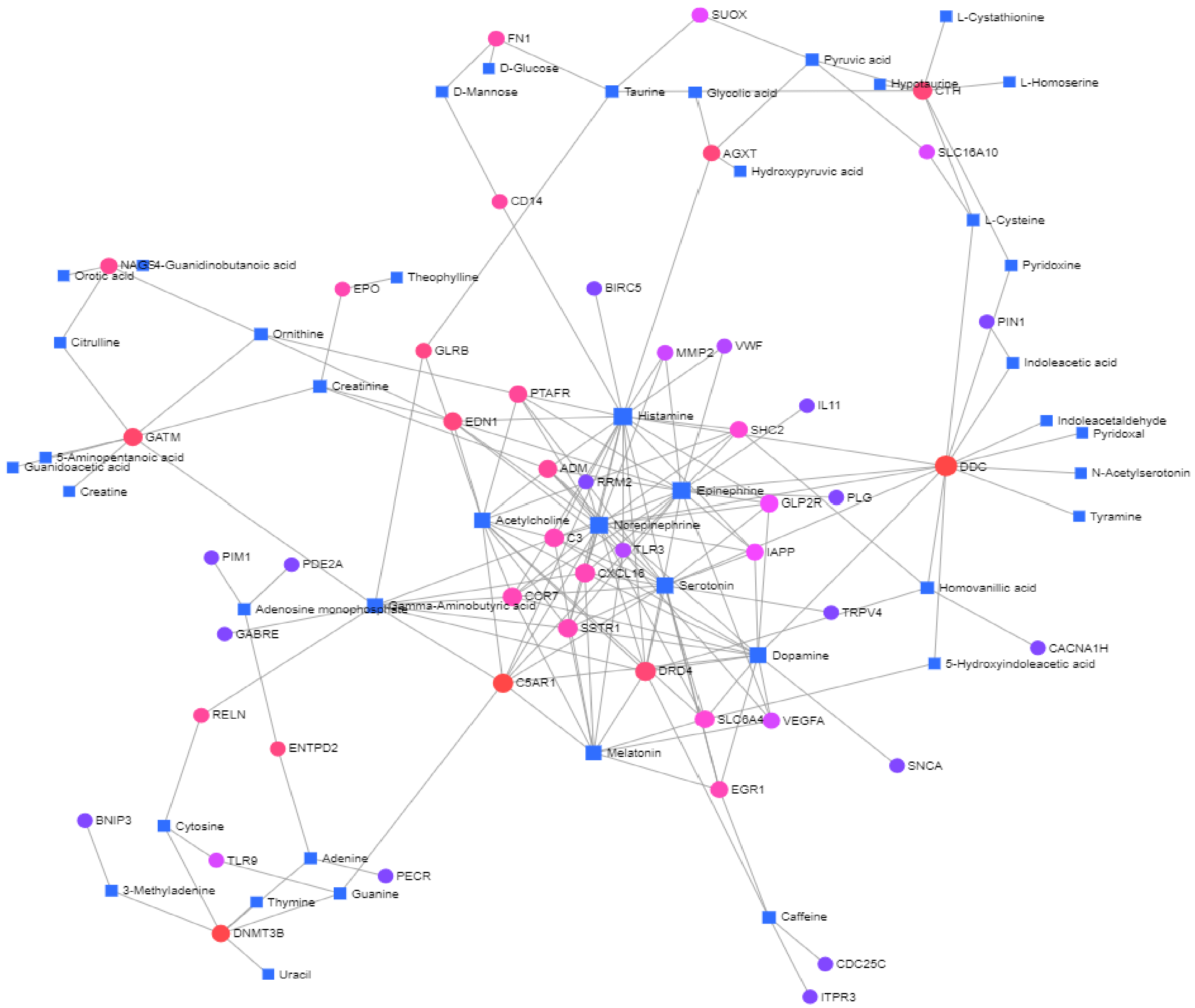

Supplemental Figure 4B. Gene-metabolite interaction maps showing the effect of ERα specific agonist PPT.

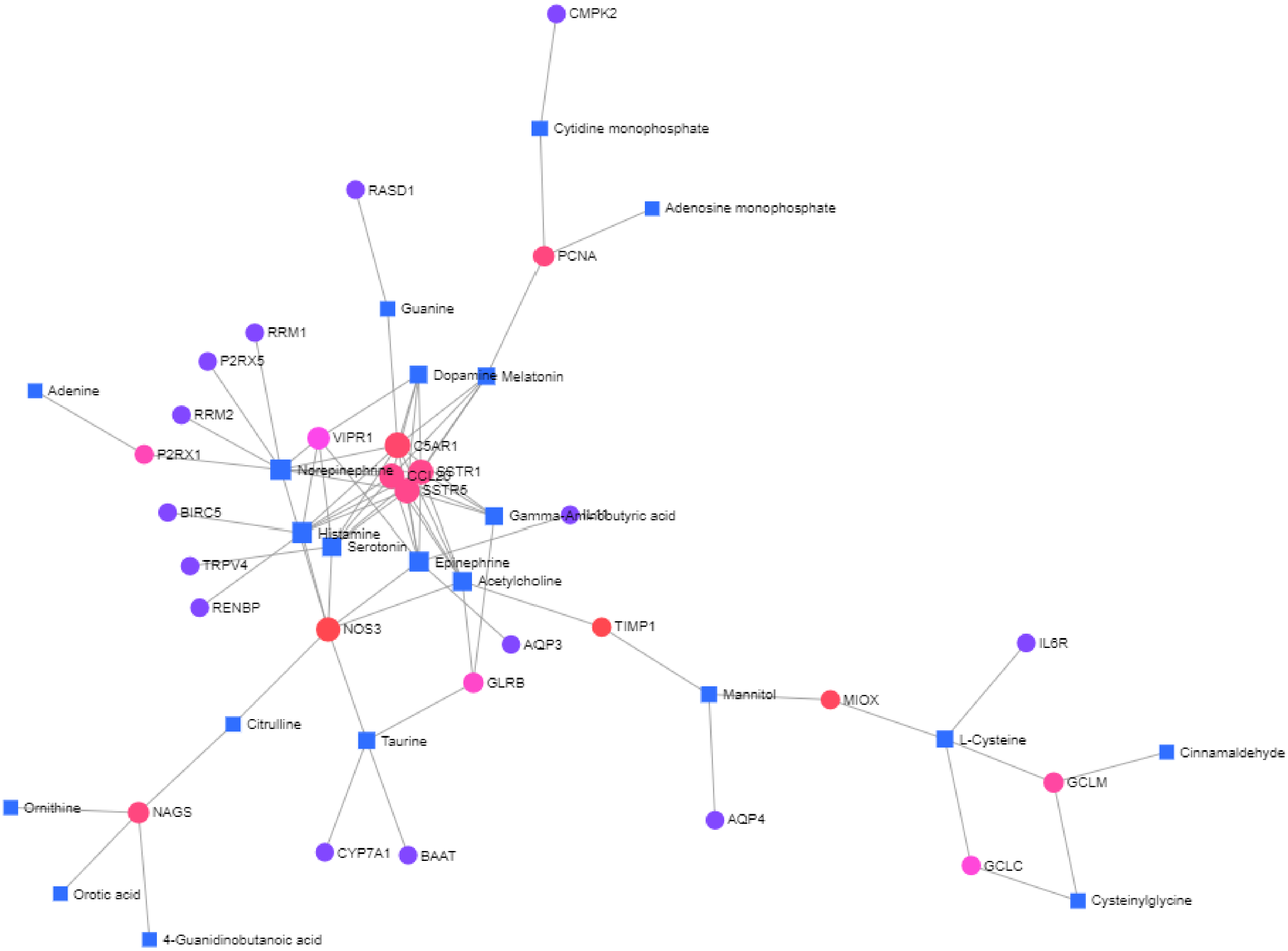

Supplemental Figure 4C. Gene-metabolite interaction maps showing the effect of ERβ specific agonist DPN.

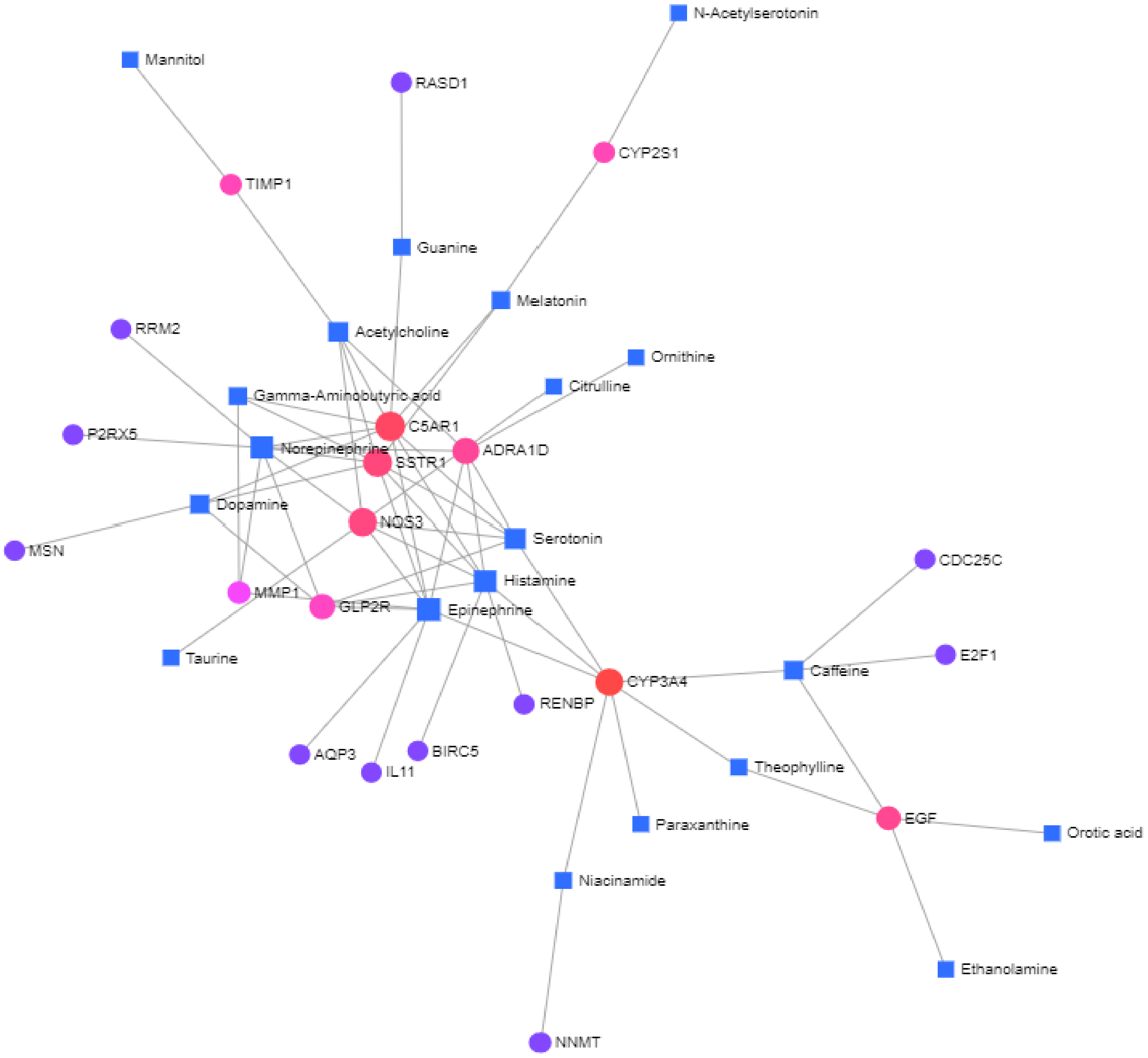

Supplement: Supplementary file 1 [file cells-10-00455-s001.pdf]
